# FunSAV: Predicting the Functional Effect of Single Amino Acid Variants Using a Two-Stage Random Forest Model

**DOI:** 10.1371/journal.pone.0043847

**Published:** 2012-08-24

**Authors:** Mingjun Wang, Xing-Ming Zhao, Kazuhiro Takemoto, Haisong Xu, Yuan Li, Tatsuya Akutsu, Jiangning Song

**Affiliations:** 1 National Engineering Laboratory for Industrial Enzymes and Key Laboratory of Systems Microbial Biotechnology, Tianjin Institute of Industrial Biotechnology, Chinese Academy of Sciences, Tianjin, China; 2 Department of Computer Science, School of Electronics and Information Engineering, Tongji University, Shanghai, China; 3 Department of Bioscience and Bioinformatics, Kyushu Institute of Technology, Iizuka, Fukuoka, Japan; 4 Bioinformatics Center, Institute for Chemical Research, Kyoto University, Uji, Kyoto, Japan; 5 Department of Biochemistry and Molecular Biology, Monash University, Melbourne, Australia; Kyushu Institute of Technology, Japan

## Abstract

Single amino acid variants (SAVs) are the most abundant form of known genetic variations associated with human disease. Successful prediction of the functional impact of SAVs from sequences can thus lead to an improved understanding of the underlying mechanisms of why a SAV may be associated with certain disease. In this work, we constructed a high-quality structural dataset that contained 679 high-quality protein structures with 2,048 SAVs by collecting the human genetic variant data from multiple resources and dividing them into two categories, i.e., disease-associated and neutral variants. We built a two-stage random forest (RF) model, termed as FunSAV, to predict the functional effect of SAVs by combining sequence, structure and residue-contact network features with other additional features that were not explored in previous studies. Importantly, a two-step feature selection procedure was proposed to select the most important and informative features that contribute to the prediction of disease association of SAVs. In cross-validation experiments on the benchmark dataset, FunSAV achieved a good prediction performance with the area under the curve (AUC) of 0.882, which is competitive with and in some cases better than other existing tools including SIFT, SNAP, Polyphen2, PANTHER, nsSNPAnalyzer and PhD-SNP. The sourcecodes of FunSAV and the datasets can be downloaded at http://sunflower.kuicr.kyoto-u.ac.jp/sjn/FunSAV.

## Introduction

With the rapid progress of genomic profiling technologies such as single nucleotide polymorphism allele genotyping arrays and next-generation DNA sequencing, an unprecedented amount of information about single amino acid variants (SAVs) has been produced. According to the recent results of the 1000 Genomes project [1], there are approximately 15 million SNPs, and 1 million short insertions and deletions, and 20,000 structural variants in the human genome [1], which are still rapidly increasing. It is estimated that there are 3∼5 million SAVs in an individual according to the recent sequencing of the whole human genome [2], [3], [4], [5].

SAVs, also known as non-synonymous SNPs (nsSNPs), are the most abundant form of single nucleotide polymorphisms (SNPs) that cause amino acid substitutions in the protein products [6]. Among various SAVs, some may cause deleterious diseases while other amino acid substitutions are neutral which will not affect the function of the protein. Previous studies on protein structures and functions have suggested that some SAVs are responsible for certain disease types, and it is reported that about 60% of Mendelian diseases are caused by amino acid substitutions [7]. The information of SAVs can be used to trace the migration patterns of ancient humans and the ancestry of modern humans. Nonetheless, its most important application may be to interpret the functional effect and impact of genomic variation, relating complex interactions with phenotypes and translating these discoveries into medical practices [8]. Therefore, discriminating disease-associated (i.e. non-neutral) from neutral variants is of great importance in the post-genomic era, which can help understand the genotype/phenotype correlations and develop treatment strategies for diseases. It is also important to identify whether a SAV is neutral or non-neutral from the disease diagnosis perspective.

In the past few decades, a variety of computational methods have been developed to predict the functional impact of SAVs in a protein [9], [10], [11], [12], [13], [14], [15], [16], [17]. These methods typically employ approaches such as statistical rules or machine learning algorithms. The input features used by these methods generally include amino acid sequence, 3D structure, physicochemical properties of amino acids, evolutionary information and complex residue-contact network features. Most of these methods have been implemented as standalone software or webservers to provide academic-free prediction of the functional impact of SAVs to the research community. Most of these methods were developed based on protein sequence analysis, such as SIFT [18], SNAP [15], PANTHER [19] and PhD-SNP [20]. The consensus of those studies is that sequence features are essential for making the prediction, while 3D structural features could further improve the prediction of disease-associated SAVs when structure information is available.

**Figure 1:**
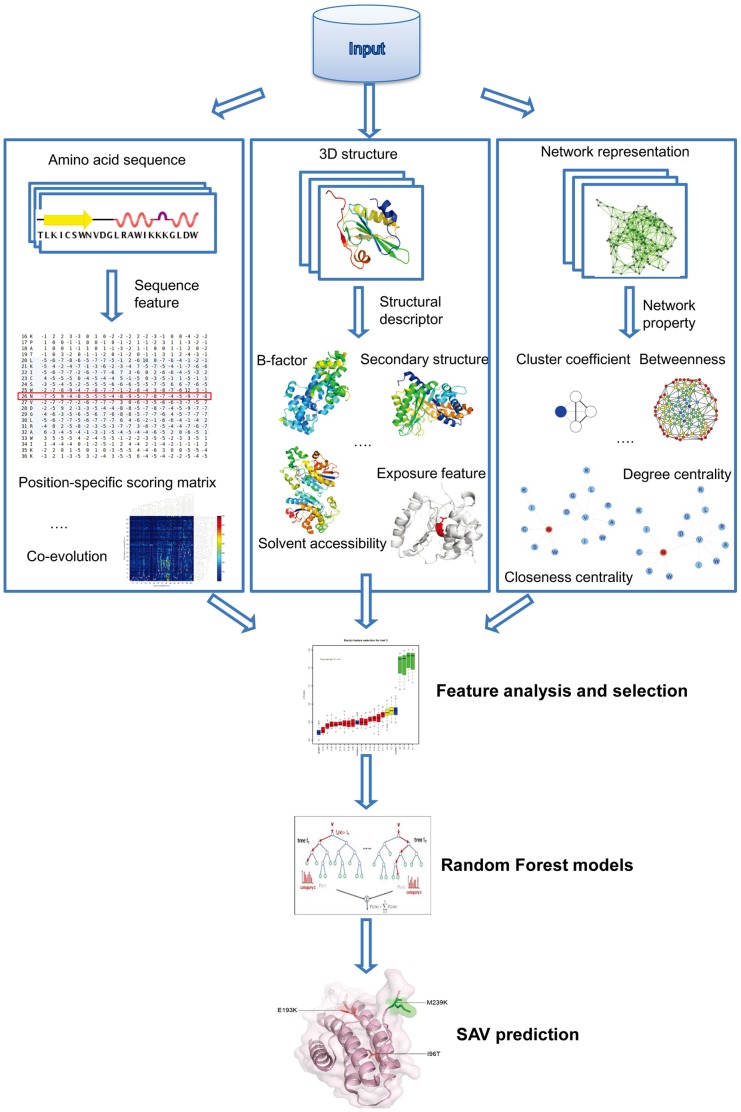
Overview of the FunSAV method for predicting the functional effect of SAVs. Features used by FunSAV are derived from the amino acid sequence of the protein, 3D structure of the protein, as well as network properties which are calculated based on the representation of the protein structure as a residue-residue contact network. A full list of the extracted features is given in Table 1. After feature selection, distinguishable features between disease-associated and neutral SAVs are statistically analyzed and used as the input to construct RF models. Prediction performance is evaluated by both 5-fold cross-validation and independent tests.

In this study, we present a novel approach for predicting the functional impact of SAVs based on a two-stage random forest algorithm. This approach, termed as FunSAV (Functional effect predictor of SAVs) (See Fig. 1 for an overview of the methodology), combines a variety of sequence and structural features as well as network properties and uses a two-step efficient feature selection to remove the noisy and redundant features in order to characterize the relative importance of each feature type. The final two-stage FunSAV classifier takes as input the prediction outputs from the first-stage classifier and scores from other prediction tools. Extensive comparisons of FunSAV with six other popular tools on the benchmark dataset and another independent test dataset show that this two-stage predictor provides a competitive performance with most of the tools, illustrating the effectiveness and advantage of this new approach.

## Materials and Methods

### Datasets

We retrieved the disease-associated and neutral SAVs to compile a structural benchmark dataset of human genetic variants [21]. First, disease-associated variants were extracted from the UniProt [22] human sequence variations (release 2010_11 as of 02 Nov 2010) where variants were divided into three categories: disease, polymorphism or unclassified. Disease-associated variants were further filtered by removing non-Mendelian disease variants that have not been assigned any MIM number from the OMIM database [23]. Neutral variants were taken from the Ensembl human variation database [24] (version 59_37d). In this study, we only extracted the verified SAVs by the HapMap project [25] to construct a high-quality benchmark dataset. Cd-hit [26] was then used to cluster protein sequences and reduce sequence homology in the dataset at the sequence identity (SI) level of 40%, in order to minimize the dataset bias introduced by homologues. All the sequences in the initial dataset were further mapped to the PDB database [27] by BLAST search [28]. All the NMR structures and the structures solved by X-Ray diffraction with resolutions lower than 2.5 Å were excluded. Details of how to map the locations of variants onto the corresponding PDB structure can be found in previous work [21]. Next, ambiguous and conflicting annotations of the disease-associated and neutral variant entries were removed. Finally, we obtained a dataset with 679 protein structures containing 1,056 disease-associated and 992 neutral SAVs, with a roughly balanced ratio of 1:1. We randomly chose 865 disease-associated and 801 neural SAVs as the benchmark dataset and the rest comprising of 191 disease-associated and 191 neutral SAVs as the independent dataset in order to validate our method.

### Feature Extraction

#### Sequence or sequence-derived features

We derived a variety of different sequence features that have proved useful in previous studies of the functional effect prediction of SAVs. These include: (1) position-specific scoring matrices (PSSMs) generated by PSI-BLAST [28]; (2) predicted secondary structure by PSIPRED [29]; (3) predicted solvent accessibility by the SSpro program from the SCRATCH package [30]; (4) predicted native disorder by DISOPRED [31]; (5) Conservation score extracted from the PSSM generated by PSI-BLAST; (6) PSIC score that represents how likely it is for a particular amino acid to occupy a specific position in protein sequence, calculated by PSIC [32]; (7) Aggregation properties calculated by TANGO [33] were used to describe the residue β-aggregation properties at mutation sites [34]. Combination of these sequence-derived features has been shown to be useful for predicting structural or functional properties of proteins in our recent work [9] and that of others [35], [36], [37], [38].

#### Structure features

We used DSSP [39] to extract the secondary structure annotations, including hydrogen bonds, solvent-accessible surface area, C_α_ atom coordinates and backbone torsion angles. The number of hydrogen bonds was calculated by HBPLUS [40].

#### Conservation score

Evolutionary conservation is an important concept in bioinformatics. Disease-related mutations are frequently observed in evolutionarily conserved positions, as these positions are essential for maintaining the structure or function of the protein [18], [41]. In contrast, neutral variants often appear in positions that have the potential to be mutated during evolution [41]. Therefore, the conservation score is a critical feature for predicting the function impact of SAV.

The conservation score can be defined as:
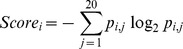
where *p_i,j_* is the frequency of amino acid *j* at position *i*. These parameters were extracted from the PSSM generated by PSI-BLAST. A lower value of the conservation score indicates a higher conservation at such position.

#### Coevolutionary features

Coevolutionary features have been recently found useful for identifying important co-evolving residues that are more likely to be disease associated upon mutation [42]. We employed several algorithms and extracted their respective coevolutionary scores as the candidate features. Among them, MI (Mutual Information) is a quantity that measures the mutual dependence between two random variables [43]. MIr [44] is a refined method that normalizes the raw MI value using the pair entropy. MIp [45] is another improved measure which removes the background MI by subtracting APC (Average Product Correction) from the original MI value. Kai is another method using chi-squared statistical methods [46] to detect residue co-evolution from sequence alignments.

#### Residue-contact network features

They were calculated as follows: Two residues in a structure will be defined as in contact if the distance between the centers of them is within 6.5 Å. Graph-theoretic approaches from the perspective of residue-residue contact networks is becoming a powerful tool to analyze and predict the functional impact of SAVs in recent years [47]. In this study, we calculated a number of distinctive residue-contact network properties that describe the local environment of the mutation residue in the residue-contact network, including degree, closeness, status, hubscore, clustering coefficient, cyclic coefficient, constraint, betweeness, eigenvector, cocitation, coreness and eccentrality.

#### Solvent accessibility

Solvent accessibility has been shown to be a powerful feature in predicting the disease association [48], [49]. Apart from the predicted solvent accessibility by SSpro from protein sequence, we also used the NACCESS program [50] to calculate the absolute and relative solvent accessibilities of all atoms, total side chain, main chain, non-polar side chain and all-polar side chain, respectively.

#### Solvent exposure features

New solvent exposure features such as Half-Sphere Exposure were used as candidate features, which were calculated by the hsexpo program [51]. These include the coordination number (CN), number of C_α_ atoms in the upper Half-Sphere (HSEAU), number of C_β_ atoms in the upper Half-Sphere (HSEBU), number of C_α_ atoms in the lower Half-Sphere (HSEAD), number of C_β_ atoms in the lower Half-Sphere (HSEBD), residue depth (RD) and atom depth (RDa).

#### Annotations from database

Annotations regarding the functional sites of a protein can be found in the “FT” line in UniProt [22]. We extracted nine different types of functional annotations: ACT_SITE, BINDING, CA_BIND, DISULFID, DNA_BIND, LIPID, METAL, NP_BIND and MOD_RES.

#### Prediction scores by other tools

These include: (i) SIFT score, which was calculated by the SIFT program that uses sequence homology to predict whether a substitution affects protein function [10], [18]; (ii) SNAP score: SNAP is a method that predicts the functional effect of single amino acid substitutions based on neural networks [15]; (iii) Polyphen2 score [52]: It is a tool based on Naïve Bayes and its output probability of being variant damaging for a SAV was used as the input feature; (iv) PANTHER score [19]: it uses Hidden Markov Models (HMMs) to predict the effect of missense SNPs on protein function and can output the probability at which a variant is deleterious; (v) nsSNPAnalyzer [53], which is based on the RF algorithm and outputs the predicted phenotypic class. We encoded the disease-associated class as 1 and neutral as -1; (vi) PhD-SNP [20], which is based on SVM [20] and outputs the predicted phenotypic class. Similarly, we encoded the predicted class into our RF models.

#### Feature vector encoding

The extracted features are listed in Table 1. We used a sliding window approach with the size of 15 residues to extract the relevant features and used them as the input to build the RF models. In terms of feature nomenclature, each residue was respectively named as V1, V2, …, V15 according to its position in the local window, while the centered residue was denoted as V8. The elements in the PSSM (with a total dimension of 15×20 = 300) were denoted as V1, V2, …, V300, respectively. Table 2 lists the abbreviations of the 15 final selected features used in this study.

**Table 1:** Features used in this study, which are categorized into nine major types: sequence or sequence-derived, structure, residue-contact network features, computed scores, annotations from database, solvent exposure features, coevolutionary features, solvent accessibilities and conservation score.

Feature type	Annotation
Sequence or sequence derived features	Mutation residue and residue neighbor in the range of window size
	Wild type residue and mutation type residue
	PSSM (PSI-BLAST [28])
	Mass weight change upon mutation
	Aggregation properties (TANGO [33])
	SCRATCH (SSpro) score [30]
	PSIPRED score [29]
	DISOPRED score [31]
	PSIC score [32]
Structure features	B-factor
	α-helix or β-bend or coil (DSSP [39])
	ACC (number of water molecules in contact with this residue *10) (DSSP [39])
	Disulfide bond and residue distance in the 3D structure
	KAPPA: virtual bond angle (bend angle) defined by the three Cα atoms of residues I−2,I,I+2 (DSSP [39])
	ALPHA: virtual torsion angle (dihedral angle) defined by the four Cα atoms of residues I−1, I, I+1,I+2.(DSSP [39])
	TCO: cosine of angle between C = O of residue I and C = O of residue I−1. (DSSP [39])
	X-CA Y-CA Z-CA: echo of Cα atom coordinates (DSSP [39])
	Number of H-bonds (HBPLUS [40])
	Metal-binding residue and the 3D distance
	Hydrogen bond (DSSP)
	Dihedral angle, C_α_ atom coordinates (DSSP [39])
	Distance between SAVs to the origin of the coordinates
Network features	degree, closeness, status, hubscore, clustering coefficient, cyclic coefficient, constraint, betweeness, eigenvector, cocitation, coreness, eccentrality.
Computed scores	SIFT score [18]
	PolyPhen2 score [52]
	SNAP score [15]
	PANTHER [19]
	nsSNPAnalyzer [53]
	PhD-SNP [20]
Annotations from database	Functional region annotation from UniProt [22]
	Sequence distance between SAV and functional region
	3D distance between SAV and functional region
	Pfam family annotation from Pfam [73]
Solvent exposure features	Solvent exposure feature calculated by biopython [51]
Coevolutionary features	MI, MIp, MIr and Kai value
Solvent accessibilities	Solvent accessibility calculated by NACCESS [50]
Conservation score	Conservation score

**Table 2:** Abbreviations of the 15 final selected features in this study.

Feature name	Residue Position	Abbreviation
The non-polar side chain solvent accessibility calculated by NACCESS	V8	NAC_npa_V8
Conservation score	V8	Con_V8
SSpro	V8	SSpro_V8
Mass weight change	–	MW_ch
PSSM	V160	PSSM_V160
B-factor	V7	B_factor_V7
Coevolutionanry feature MI	V8	Co_MI_V8
Exposure feature HSEBD	V8	HSEBD_V8
Exposure feature RD	V8	RD_V8
Exposure feature HSEBU	V9	HSEBU_V9
Exposure fature CN	V9	CN_V9
Network feature Status	V1	Status_V1
Network feature Closeness	V7	Closeness _V7
Network feature Status	V9	Status_V9
Network feature Status	V7	Status_V7

### Performance Evaluation

We used Sensitivity (SEN), Specificity (SPE), Precision (PRE), Accuracy (ACC), the Matthew’s correlation coefficient (MCC) and the area under the curve (AUC) to evaluate the predictive performance of our method.

The Sensitivity (SN) is defined as:
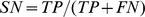



The Specificity (SP) is defined as:
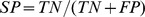



The Precision (PRE) is defined as:
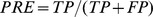



The overall Accuracy (ACC) is defined as:
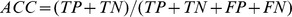



The Matthew’s correlation coefficient (MCC) [54] is defined as:
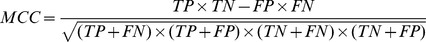
where *TP* is the number of true positives, *TN* is the number of true negatives, *FP* is the number of false positives and *FN* is the number of false negatives, respectively.

More specifically, AUC is the area under the receiver operating characteristic (ROC) curve, which is a plot of true positive rate (TPR) against false positive rate (FPR). TPR is the ratio of the number of correctly classified disease-associated SAVs to the total number of disease-associated variants, while FPR is the ratio of the number of correctly classified neutral SAVs to the total number of neutral variants.

### Feature Selection

We proposed a novel two-step feature selection procedure to select the most informative features for predicting the functional effect of SAVs. The first feature selection method is based on the mean decrease Gini index (MDGI), which was calculated by the RF package in R [55]. MDGI is the mean decrease of Gini index, which is equal to the Gini coefficient multiplied by 100. The Gini coefficient is a measure of inequality of a distribution and is defined as a ratio of the areas on the Lorenz curve diagram [56]. MDGI represents the importance of individual vector element for correctly classifying a SAV as being disease-associated or neutral. The mean MDGI Z-Score of each vector element is defined by the following equation:
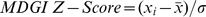
where *x_i_* is the mean MDGI of the *i*-th feature, 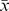 is the mean value of all elements of the feature *x* and *σ* is the standard deviation (SD), respectively. In this study, the vector element with MDGI Z-Score larger than 1.0 was selected as an optimal feature candidate (OFC) determined by the MDGI Z-Score.

The second step is a stepwise feature selection by training and evaluating the corresponding RF classifiers based on five-fold cross-validation tests. We randomly divided our benchmark dataset into five subsets in each validation step. Then at each cross-validation step, four subsets were merged as the training set to train the model, while the rest subset was singled out as the test set to validate the built model. This procedure was repeated five times such that each subset was used in the training and validated in the testing. Then the above five-fold cross-validation procedure was repeated 100 times. As a result, we calculated the average of predicted scores of RF classifiers, and then carried out the performance evaluation.

We performed the stepwise feature selection (also called backward feature selection) by training the RF model with all the initial OFC features in the first round. Then in the second round, one feature would be removed from the initial feature set. In this round, each feature would be removed once a time, and all of the 65 combinations (each containing the rest 64 features) were used to train the corresponding RF models whose performance would be subsequently evaluated. If the resulting RF predictor achieved a higher MCC, such feature would be removed and the corresponding combination was used in the next round. This stepwise feature selection process continued until MCC no longer increased. In this way, most important and informative features can be systematically identified.

### Random Forest

The random forest algorithm was originally developed by Leo Breiman [57] and has been implemented as the Random Forest package in R [55]. In this study, we designed and constructed the first-stage and two-stage RF models of FunSAV. Specifically, the first-stage RF classifier of FunSAV was trained based on the optimal 15 features that were selected through a two-step feature selection procedure. We further developed a two-stage predictor, which was trained using RF by combining the outputs of the first-stage classifier and the scores from six other tools SIFT, SNAP, PolyPhen2, nsSNPAnalyzer, PANTHER and PhD-SNP.

## Results and Discussion

### Optimal Feature Candidate Selection

It is well known that efficient feature selection can significantly improve the prediction performance of machine learning-based classifiers. Furthermore, feature selection can be used to select the most relevant and informative features that contribute to the success of a classifier by reducing the initial high-dimensional feature space to a lower but more compact one. In this work, we selected 15 optimal features that were shown to better distinguish disease-associated from neutral SAVs to train the first-stage FunSAV classifier based on the constructed benchmark dataset.

**Figure 2:**
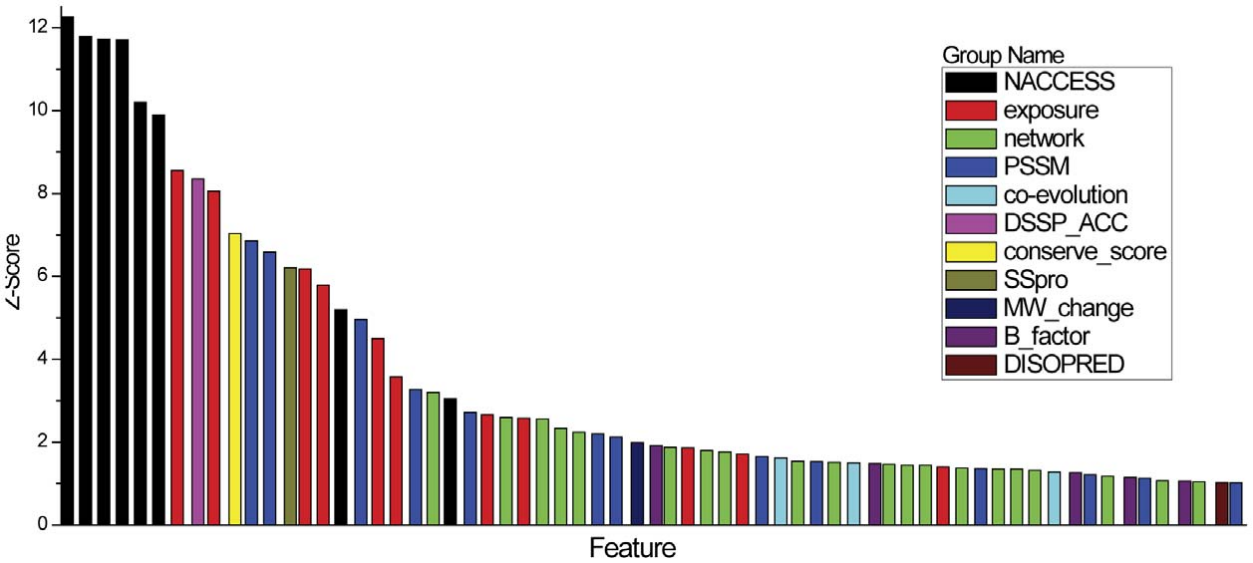
The relative importance and ranking of the optimal feature group, as evaluated by the mean MDGI Z-Score. The bar represents the mean MDGI Z-Score of the corresponding feature group. NACCESS: solvent accessibilities calculated by NACCESS [50]; exposure: solvent exposure features calculated by the biopython package [51]; network: residue-contact network features calculated by the JUNG library available at http://jung.sourceforge.net/; PSSM: PSSM features calculated by PSI-BLAST [28]; co-evolution: coevolutionay features including MIr, MIp, MI and Kai value; DSSP_ACC: the number of water molecules in contact with the residue of interest extracted from DSSP [39]; conserve_score: conservation score defined in the Feature extraction Section; SSpro: solvent accessibility calculated by the SSpro program [30]; MW_change: Mass weight change upon mutation; B_factor: the temperature factor extracted from the PDB file; DISOPRED: predicted native disorder by DISOPRED [31].

These 15 final optimal features were selected by two consecutive steps. In the first step, the mean MDGI Z-Scores of all the 1804 initial features (see Table S1 for a full list) were calculated by RF and the relative importance of these features was sorted and evaluated. As a result, 65 features with the mean MDGI Z-Score >1.0 were selected as OFCs. The relative importance and ranking of the optimal feature groups are given in Figure 2. Among them, the feature with the highest mean MDGI Z-Score (>9.0) is the solvent accessibility feature calculated by NACCESS. Solvent exposure features and DSSP_ACC also have larger MDGI Z-Scores, while network and co-evolution features have moderate MDGI Z-Scores ranging from 1.0 to 4.0 (Fig. 2).

The second step is to further select more important features stepwisely. Figure S1 shows the performance of RF-based classifiers in terms of MCC by gradually incorporating stepwise selected features. The mean values, standard deviations of the 15 finally selected features and the *P*-values indicating the statistical significance between the disease-associated and neutral SAVs are provided in Table S2. It can be seen that four types of residue-contact network features and four types of solvent exposure features were included in the final feature set. The majority of the finally selected features are descriptors of the centered mutation residue (denoted as V8), including the solvent accessibility calculated by NACCESS, conservation score, SSpro (i.e. binary classification of relative solvent accessibility as exposed or buried), exposure_HSEBD and exposure_RD. Nevertheless, other features that describe the neighboring residues of the mutation position were also included in the final feature set. These include network_status_V1, network_status_V7 and network_status_V9, where V1, V7 and V9 denote neighboring residues at positions surrounding the centered mutation residue V8. These indicate that descriptors of neighboring residues of the variants also play an important role in discriminating disease-associated from neutral SAVs.

Our two-step feature selection is similar to that of Ebina *et al*. [58]. The major difference is that they used SVM in the first step and RF in the second step to build their classifiers, whereas we used RF consistently in both steps. Another difference is that they removed or added individual features by dividing OFC into two subsets and examining the resulting performance of the classifiers, while we performed a less time-consuming backforward feature selection from the whole set of OFCs in the second step. Generally speaking, this two-step feature selection has two attractive advantages: (1) It provides a realistic way for selecting an optimal subset of features with an acceptable computational burden [58] compared with other computationally intensive feature selection methods. The latter often rely on trial and error experiments to select the most relevant features from a relatively small set of arbitrarily selected features; (2) Although the stepwise feature selection does not necessarily require an exhaustive search and may overlook certain effective combinations of candidate features, it manages to evaluate a sufficient number of feature combinations and results in one of the best combinations.

We also assessed the prediction performance by combining different feature groups that correspond to different ranges of MDGI Z-scores. The resulting performances are given in Table S3. Our stepwise feature selection was performed using a set of 65 features with Z-Score>1.0. After feature selection, the RF classifier based on the final optimal feature set attained the MCC value of 0.510 from the initial value of 0.458. In the meanwhile, the number of selected features decreased from 65 to 15. The results indicate that stepwise feature selection is effective at identifying more important and informative features. After the removal of redundant and less informative features with feature selection based on the Z-scores, we can efficiently improve the performance of the RF-based classifiers.

### Feature Importance and Contribution

**Figure 3:**
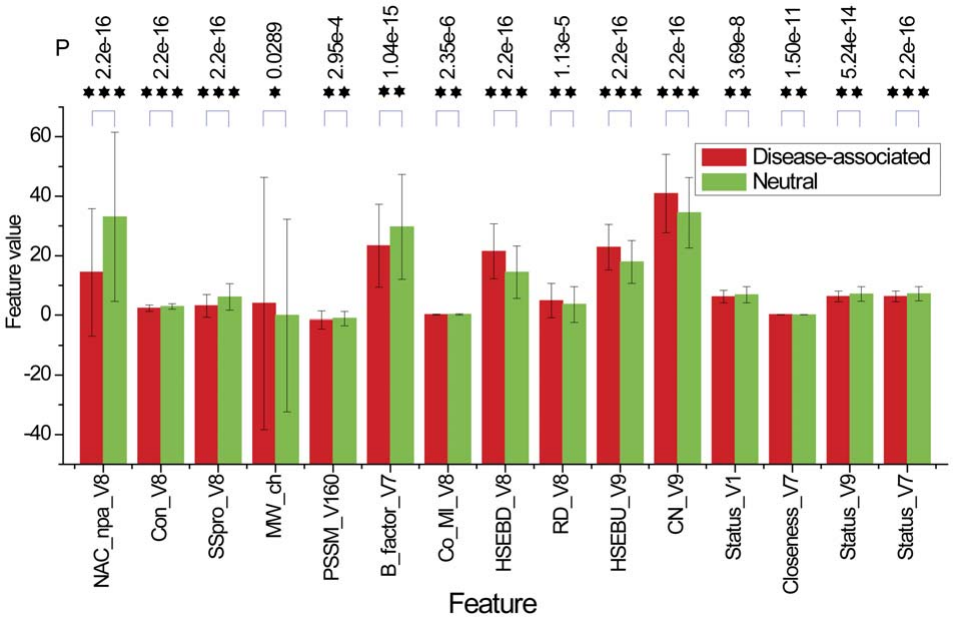
Comparison of the mean values and standard deviations of the 15 optimal features of disease-associated and neutral SAVs. “*” represents a *P*-value in the range of 0.01∼0.05, “**” represents a *P*-value in the range of 2.2e-16∼0.01, while “***” represents a *P*-value<2.2e-16, respectively. See Table 2 for more details about feature abbreviations.

In this section, we elaborated on the 15 finally selected optimal features. We compared the MDGI Z-scores of these features and performed the unpaired two-sample *t*-test (Table S2). The *t*-test is a statistical test of whether the mean values of a given feature between the two sources (i.e. disease-associated and neutral SAVs) are equal and thus evaluates the potential of such feature in the discrimination of the two sample sets. The results are illustrated in Figure 3. It can be seen that for most of the selected features, the mean values between the disease-associated and neutral SAVs are significantly different, with the *P*-value <<0.01. The only exception is that the MW_change feature has a *P*-value of 0.0289.

**Figure 4:**
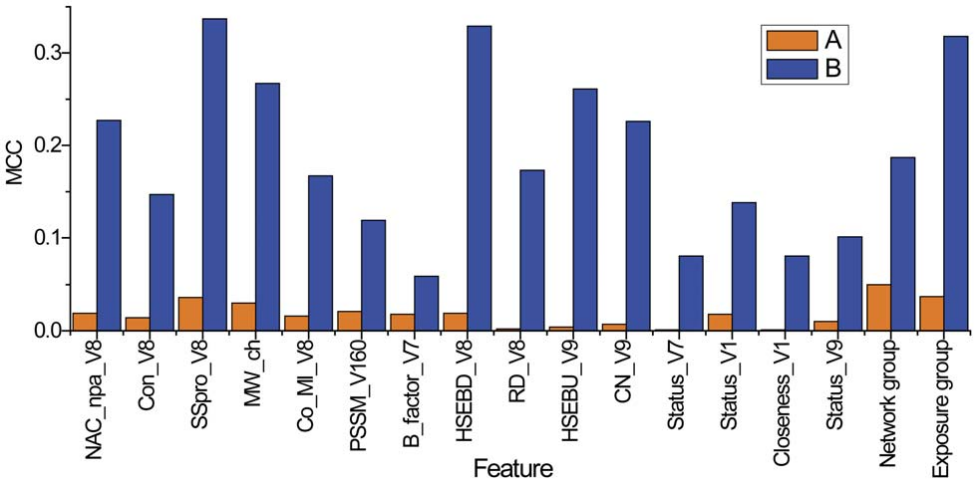
Effect of the removal or inclusion of the 15 individual optimal features on the prediction performance of the first-stage FunSAV classifier. Performance was evaluated using MCC. A: Performance of the trained classifier using the individual feature; B: MCC decrease of the trained classifier by removal of the corresponding feature. See Table 2 for more details about feature abbreviations.

Previous studies have found solvent accessibilities to be powerful features for improving the performance [34]. In this study, we confirm that the most important and contributive features are related to solvent accessibility, including the solvent accessibility feature calculated by NACCESS, DSSP_ACC, solvent exposure features, and the SSpro score which is a descriptor of binary burial status. We note that most features with higher Z-Scores belong to the solvent accessibility feature group (Fig. 2). Among them, SSpro score is the most important feature in terms of the contribution to the performance improvement (See Table S4 and Fig. 4). If the SSpro feature was removed from the final feature set with 15 optimal features, the MCC of the resulting classifier would dramatically decrease from 0.510 to 0.474. Moreover, the classifier that was trained using only the SSpro feature achieved an MCC of 0.337, which is the highest value among all the individual classifiers trained based on singular optimal features (Table S4). This observation is consistent with previous studies that suggest disease-associated SAVs were more frequently observed in buried sites [59]. We find that for disease-associated SAVs this feature is significantly different from that of neutral SAVs (*P*-value<2.2e-16). Although SSpro primarily predicts solvent accessibility from sequences information, its prediction performance has also benefited from the incorporation of high-quality structural templates [30]. Therefore, the prediction of SSpro essentially relies on an effective combination of both the complementary sequence and structural information. This is particularly advantageous and has an important implication for improving the training quality of machine learning predictors to learn the complex sequence-structure-function relationship of proteins. Thus, inclusion of this feature in the classifier is useful for improving the performance. Further analysis of solvent accessibility features calculated by NACCESS revealed a different tendency of disease-associated and neutral SAVs. Neutral SAVs have higher NACCESS scores on average than disease-associated SAVs (Fig. 3). This means that disease-associated variants are more likely to occur at positions with lower solvent accessibility compared with neutral variants, that is, they tend to be relatively deeply buried in the structure. We calculated the solvent accessibilities of the total (all atoms), non-polar side chain, polar side chain, total side chain and main chain using NACCESS [50]. After stepwise feature selection, only the solvent accessibility feature of the non-polar side chain was retained in the final feature set, which was calculated based on all non-oxygens and non-nitrogens in the side chain. The solvent accessibility of all non-oxygens and non-nitrogens in the side chain is more important than other solvent accessibility features. A possible explanation is that the atoms (the oxygen or nitrogen) in the side chain play an important role in forming interactions with other residues of the protein and the water molecules. These interactions among the side-chain atoms, other residues and solvent molecules are often critical for the functionality of the protein.

We noticed several solvent exposure features that were not employed in previous studies but were found to be useful for the prediction. These include four solvent exposure features selected in the final optimal feature set, including HSEBD, RD, HSEBU and CN. These features have distinctive distribution tendencies between disease-associated and neutral SAVs, i.e. higher for disease-associated SAVs and lower for neutral SAVs (Fig. 3). For example, disease-associated SAVs have relatively higher RD values, which means that they are more likely to appear in the inner layer of the protein. CN is a feature that calculates the number of C_α_ atoms within a sphere around the C_α_ atom of the centered residue, which has been shown to be correlated with the change in protein stability (measured by the free energy of unfolding) [51]. HSEBU and HSEBD were calculated by dividing the sphere into two half spheres and subsequently counting the numbers of neighboring residues in each half sphere. Although these four features have an inter-correlation, they have a good complementarity and thus collectively make a contribution to the performance improvement.

**Table 3:** Prediction performance of the first-stage and two-stage FunSAV classifiers in comparison with six other prediction tools.

Classifier	Performance					
	MCC	ACC	SEN	SPE	PRE	AUC
SNAP	0.426	0.680	0.932	0.441	0.612	0.740
SIFT	0.475	0.734	0.806	0.665	0.695	0.807
PolyPhen2	0.512	0.745	0.879	0.618	0.685	0.838
nsSNPAnalyzer	0.334	0.665	0.546	0.778	0.699	0.662
PANTHER	0.500	0.749	0.776	0.724	0.727	0.816
PhD-SNP	0.350	0.676	0.653	0.697	0.671	0.675
First-stage classifier	0.535	0.767	0.772	0.763	0.755	0.824
PolyPhen2+SIFT+SNAP+nsSNPAnalyzer+PANTHER+PhD-SNP	0.513	0.757	0.802	0.708	0.748	0.831
Two-stage classifier	0.598	0.799	0.797	0.801	0.792	0.882

Another important feature that was not found useful in previous studies is co-evolution. It refers to a phenomenon induced by the demand of maintaining the structure and/or function of a protein during its evolution. We find that neutral SAVs have relatively higher co-evolution values than disease-associated SAVs. This suggests that neutral variants are more likely to be involved in co-evolution, while disease-associated SAVs are more conserved in the evolution process. The MCC of the classifier trained using the co-evolution feature is 0.167, and the MCC decrease of the resultant classifier after removal of this feature is 0.016 (Table S4), which is a moderate decrease compared to the other 14 individual classifiers. This shows that co-evolution is also is a relatively important feature to distinguish disease-associated SAVs from neutral SAVs.

**Figure 5:**
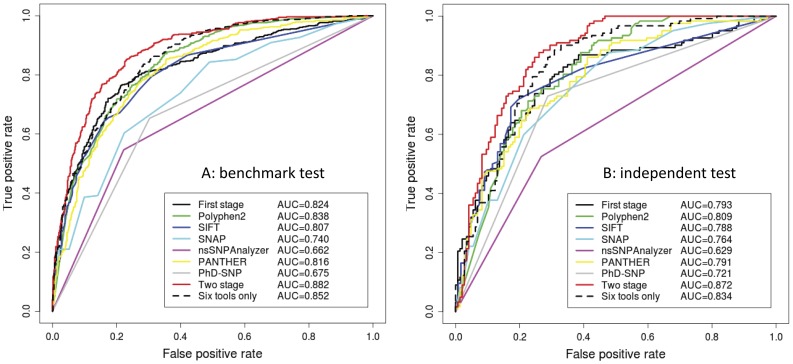
The ROC curves of nine classifiers based on 5-fold cross-validation tests. Results are evaluated based on the benchmark dataset (A) and independent test dataset (B).

The B-factor of protein crystal structures is a feature that tends to be overlooked in the functional effect prediction of SAVs. In this work, the B-factor was selected in the final subset of 15 features from the initial 1804 features. It reflects the fluctuation of atoms about their average positions and contains important information about protein dynamics [60]. It can be seen from Figure 3 that the B-factor of V7 position is significantly different between disease-associated and neutral SAVs. The neutral SAVs have higher B-factor values than the disease-associated SAVs, suggesting that the V7 position of neutral SAVs fluctuate more than disease-associated SAVs. There may be one possible reason to explain this. Since proteins are composed of consecutive polypeptide backbones and V7 position is very close to the V8 position where the mutation actually takes place, the fluctuation of V7 position also reflects the fluctuation of V8 position and hence was selected as one of the important final features. However, the reason why the position is V7 rather than V9 is not clear to us. It may be because that the C_α_ atom of V8 residue is closer to V7 than V9 residue, as the lengths of the C_α_-N and C_α_-C bonds are 0.145 and 0.152 nm, respectively. Therefore, C_α_ is closer to V7 than V9, and as a result the V7 position has a greater influence on the variant than the V9 position. Our study also revealed the significance of residue-contact network features for predicting the functional effect of SAVs. A number of features have been previously used to predict disease-associated SAVs, such as degree, clustering coefficient, betweenness and closeness [47]. Here, we included and examined more residue-contact network features. After feature selection, four such features, i.e. Status.V1, Status.V7, Status.V9 and Closeness.V7 were selected in the final feature set of 15 optimal features. They belong to two generic categories of network properties: clossness and status. Both describe the geodesic distances between the vertex of interest and all other vertices within the residue-contact graph of a protein chain.

**Figure 6:**
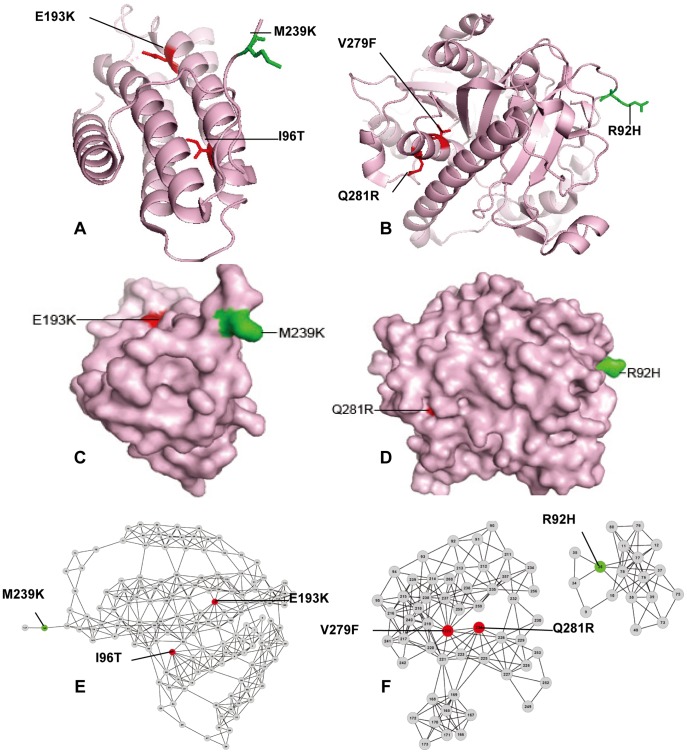
Prediction examples of the functional effect of SAVs in two proteins by FunSAV. (A) and (B) the all-atom; (C) and (D) surface; (E) and (F) network representations of proteins hATR (PDB ID: 2IDX, chain A) and PAF-AH (PDB ID: 3D59, chain A), respectively. Red color denotes disease-associated variants while green color represents neutral variants. 3D structures were rendered using PyMol [71] and network graphs were drawn using Cytoscape [72].

More specifically, closeness is a centrality measure of a vertex which describes the status of a residue located in the entire protein structure [47] where highly central residues have higher closeness values [61]. Such residues interact with a large number of other residues. Previous studies show that closeness can be effectively used to identify functionally important residues [61], [62] and disease-associated SAVs can be identified by a higher closeness measure [47]. Our results in this study are in good agreement with these studies. Nevertheless, we find the status to be an additional useful feature for the prediction, which was not previously recognized. It represents the sum over all geodesic distances between the residues of interest and all other residues in the residue-contact graph. From Figure 3, we can see that neighboring residues of disease-associated SAVs including V1, V7 and V9 have on average lower status values than neutral SAVs. The relationship between clossness and status can be expressed as 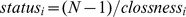, where *N* is the number of edges within the residue-contact graph. As the disease-associated SAVs can be identified by a higher closeness measure, this means that they have lower status values than neutral SAVs. From the definition of status (
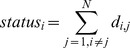
), we can see that the status value is determined by two important factors: the distance between the residue *i* and *j*, and the number of residues (i.e. *N*) in the residue-contact graph. In other words, the reason why neighboring residues of disease-associated SAVs have lower status is because either (1) the distance between the contacted residues is shorter than that of neutral SAVs neighbors; (2) the neighboring residues of disease-associated SAVs are located on the periphery of the structure and accordingly have a smaller *N* and thus a smaller status. Altogether, the closeness and status features of neighboring residues of the mutant residues (such as V7, V9 and V1 positions) were selected as important residue-contact network features in the final feature set. The reason why only the network properties of neighboring residues rather than the mutant residue itself were selected might be that these residue-contact network features reflect the interactions between different neighboring residues surrounding the centered residues and they can provide sufficient information of the critical local microenvironment of the mutant residue to improve the performance of RF classifiers.

**Figure 7:**
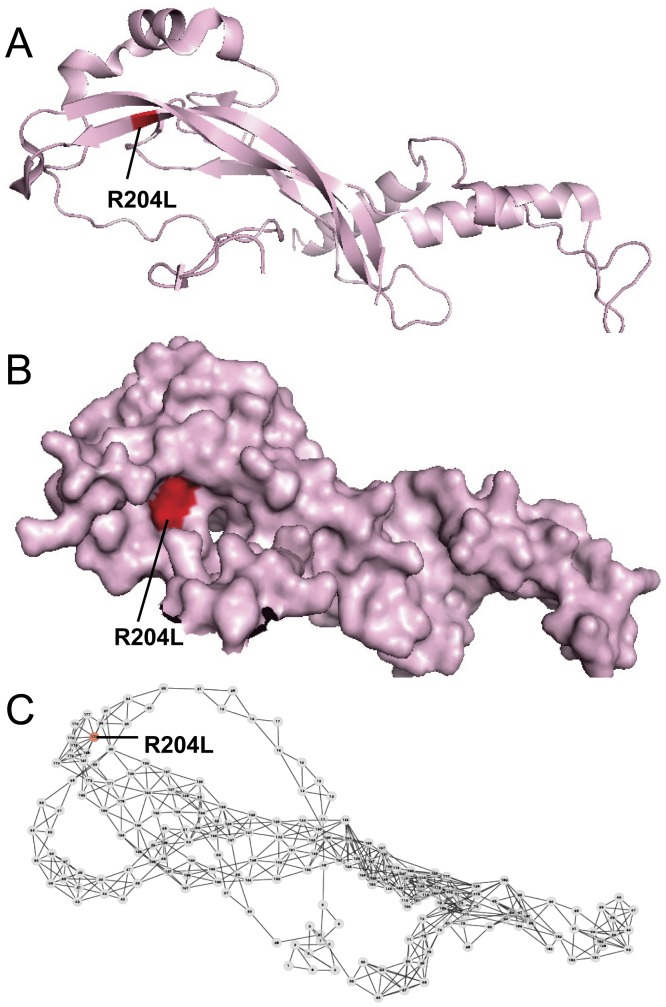
Prediction example of the false negative of the functional effect of SAVs by FunSAV for the Noggin protein. (A) The all-atom; (B) surface; (C) network representations of the Noggin protein. Red color denotes the disease-associated variant. 3D structures were rendered using PyMol [71] and network graphs were drawn using Cytoscape [72].

### Prediction Performance of FunSAV Classifiers

In this study, we chose to use RF instead of SVM as the classifier in that RF has been shown to outperform SVM in the prediction of functional impact of SAVs [63], [64] and RF classifiers do not involve time-consuming parameter optimization process and is thus much faster to train the classifiers than SVM. The 15 optimal features were used to build the first-stage FunSAV classifier, which produced a probability score of a SAV being disease associated or not. This score was then combined with the prediction scores from six other popular tools SNAP, SIFT, PolyPhen2, nsSNPAnalyzer, PANTHER and PhD-SNP, and used as the input to train the two-stage classifier. As some variant data could not be predicted by PANTHER or nsSNPanalyzer in the analysis, we performed 5-fold cross-validation tests and evaluated the performance of each of the classifiers using a subset of the benchmark dataset for which PANTHER and nsSNPanalyzer generated valid predictions (See Table 3 and Fig. 5A). The first-stage classifier achieved the highest MCC of 0.535 compared with other individual classifiers and the second highest AUC of 0.824, which is only lower than PolyPhen2 and is better than other five tools. Both PolyPhen2 and our method used structural features. These results indicate that when structure is available, incorporation of structural features are critical for improving the performance of predicting functional impacts of SAVs.

We built an integrated classifier by combining the prediction scores of the six tools (PolyPhen2+SIFT+SNAP+nsSNPAnalyzer+PANTHER+PhD-SNP) and achieved an MCC of 0.540 and an AUC of 0.852. Moreover, we incorporated the prediction output of the first-stage FunSAV classifier with prediction scores from SIFT, SNAP, PolyPhen2, nsSNPAnalyzer, PANTHER and PhD-SNP to build a two-stage FunSAV classifier. As a result, the prediction performance was significantly improved, with MCC increased from 0.535 to 0.598, and AUC from 0.824 to 0.882, respectively. Although the SNAP, SIFT and PolyPhen2 achieved higher sensitivity (93.2, 80.6 and 87.9% for SNAP, SIFT and PolyPhen2, respectively), they had lower specificity (44.1, 66.5 and 61.8%, respectively). In contrast, the first-stage FunSAV classifier achieved a balanced sensitivity and specificity (77.2 and 76.3%, respectively), while the two-stage FunSAV classifier achieved a sensitivity of 79.7% and a specificity of 80.1%, respectively. We also evaluated the prediction performances of the first-stage and two-stage FunSAV classifiers based on another independent test dataset. The results are given in Table S5 and Figure 5B. We built the final FunSAV classifier by combining the first-stage classifier with the scores of all six other tools SIFT, SNAP, PolyPhen2, nsSNPAnalyzer, PANTHER and PhD-SNP. The prediction performance of this new classifier is more robust compared to other classifiers, and has outperformed the first-stage FunSAV classifier and the other six individual tools on the independent test dataset. As a result, AUC accordingly increased from 0.793 to 0.872, and MCC increased from 0.482 to 0.606, both of which are the overall best performance.

### Case Study

To further illustrate the effectiveness of FunSAV for identifying disease-associated from neutral variants, we present a case study of three proteins that contain both disease-associated and neutral variants in this section. The first two proteins tested are not present in our benchmark dataset for building the FunSAV classifiers. FunSAV correctly identified the functional effect of all the variants in the first two proteins. The third protein is provided as an illustration of the false negatives generated by FunSAV.

The first example is the human ATP: cobalamin adenosyltransferase (hATR) [65]. This enzyme catalyzes the final step in the conversion of vitamin B12 to the human cofactor adensosylcobalamin. Mutations in hATR result in the metabolic disorder, known as methylmalonic aciduria (MMA). The variant M239K (dbSNP: rs9593) is a neutral substitution, while the variants E193K and I96T result in methylmalonic aciduria (MMA), an inborn error of metabolism due to the impaired isomerization of L-methymalonyl-CoA to succinyl CoA during the oxidation of propionate towards the TCA cycle [66]. From Figure 6A, we can see that M239 is relatively exposed at the surface of the protein, while E193K and I96T are relatively buried in the structure. Table S6 also indicates that disease-associated variants are located in buried area. The neutral variant M239 has a fewer number of interacting residues, while the disease-associated variants E193K and I96T have more densely connected edges with other neighboring residues in residue-contact network (Fig. 6E). Hence, mutations at these positions tend to disrupt the local residue-contact network and thus are more likely to cause disease.

The second example is the human plasma platelet-activating factor (PAF) acetylhydrolase (PAF-AH) [67]. It reduces PAF levels by functioning as a general anti-inflammatory scavenger and is linked to anaphylactic shock, asthma, and allergic reactions. The variants (V279F: dbSNP: rs16874954 and Q281R) will result in a loss of plasma PAF-AH activity that accounts for 4% of the Japanese population. The polymorphic site R92H is a neutral variant, which upon mutation is more likely to exhibit phenotypic differences through interactions with lipoproteins or other binding partners [67]. Similar to the variants in the above example, the variant R92H is also solvent-exposed and located in the outer layer of the structure (Fig. 6B), with fewer interactions with other residues compared with the other two disease-associated variants V279F and Q281R. The latter two variants are deeply buried in the inner layer of the structure and accordingly form highly connected residue-interacting networks. These case studies suggest that FunSAV is an effective tool for identifying functional impacts of SAVs.

The third example is the Noggin whose primary physiological role is to antagonize the action of bone morphogenetic proteins (BMP) [68]. The antagonist Noggin can bind to BMP and inhibit BMP signaling by blocking the molecular interfaces of the binding epitopes. The residue R204 of Noggin can form ion pairs with E48 of BMP. The variant R204L will cause tarsal/carpsal coalition syndrome (TCC), because it disrupts the ion pair with E48 of BMP. From Figure 7, it can be seen that the variant R204L was located at the surface of Noggin, and the values of some important features selected in the final feature set are more close to the mean of neutral SAVs (listed in Table S2) than that of disease-associated SAVs, for example, the exposure features (See Table S6 for more detail). In such cases, it would be more difficult for FunSAV to correctly predict its functional effect, while other software such as SIFT, SNAP, PolyPhen2, nsSNPAnalyzer, PANTHER and PhD-SNP could correctly predict the functional impact of this variant. Therefore, inclusion of more relevant features that describe the interactions of the protein of interest with other interaction partners may prove to be an effective way to further improve the performance of FunSAV.

### Conclusions

We developed FunSAV, a new bioinformatics tool based on the random forest algorithm to predict the functional effect of SAVs. Extensive 5-fold cross-validation and independent tests demonstrate that FunSAV has achieved a better performance compared with six other competitive tools. The performance improvement of FunSAV can be attributed to the combination of four critical factors: (i) use of high-quality balanced structural dataset; (ii) classifier trained based on a large feature set with a variety of important and complementary features, including sequence, structure, network and other types of features that describe the local environments proximal to the centered variant and neighboring residues; (iii) efficient feature selection to remove noisy and redundant features to prevent overfitting and (iv) training of robust two-stage RF classifiers in combination with scores by other tools. We show that it is especially useful to build better classifiers with improved performance through efficient feature selection from a large initial set of various features, and integration with scores by other tools. To make an accurate prediction, FunSAV requires the 3D structure of the protein where SAVs were located, which may limit its broader application. However, with the increasing availability of target structures solved by structural genomics initiatives, genome-wide protein 3D modeling projects [69] and predicted 3D structures [70], it is expected that FunSAV can be used as a powerful tool to prioritize the disease-associated variants and help towards the functional annotation of these targets.

## Supporting Information

Figure S1 **The feature selection curve in stepwise feature selection describes the performance change (in terms of MCC) of gradual inclusion of individual features to the trained classifiers.** MCC_FSS (feature selection stepwise, FSS) indicates the MCC change in this stepwise feature selection process.
(TIF)

Table S1 **All initial 1804 features used in this study.** “OFC” indicate that such feature was selected as the 65 optimal feature candidates (OFCs), while “FINAL” indicates that such feature was selected as one of the 15 final optimal features.
(DOC)

Table S2 **The mean values and standard deviations of the 15 final selected optimal features for the disease-associated and neutral SAVs.** Mean: mean value; SD: standard deviation. P-value was calculated using the unpaired two-sample *t*-test.
(DOC)

Table S3 **The prediction performance of RF-based classifiers based on different feature group combinations according to the MDGI Z-Score.**
(DOC)

Table S4 **The importance and contributeon of the 15 final optimal features by removal or inclusion of this feature to the first-stage RF classifier.**
(DOC)

Table S5 **Performance of the first-stage and two-stage classifiers based on an independent test dataset.**
(DOC)

Table S6 **Analysis of several important final features that are related to the residue locations in three exemplar proteins in the case study.**
(DOC)
