# Supplementary figures and images for: FunSAV: Predicting the Functional Effect of Single Amino Acid Variants Using a Two-Stage Random Forest Model
Source: PLoS One. 2012 Aug 24;7(8):e43847. doi: 10.1371/journal.pone.0043847 (PMC3427247; doi:10.1371/journal.pone.0043847)

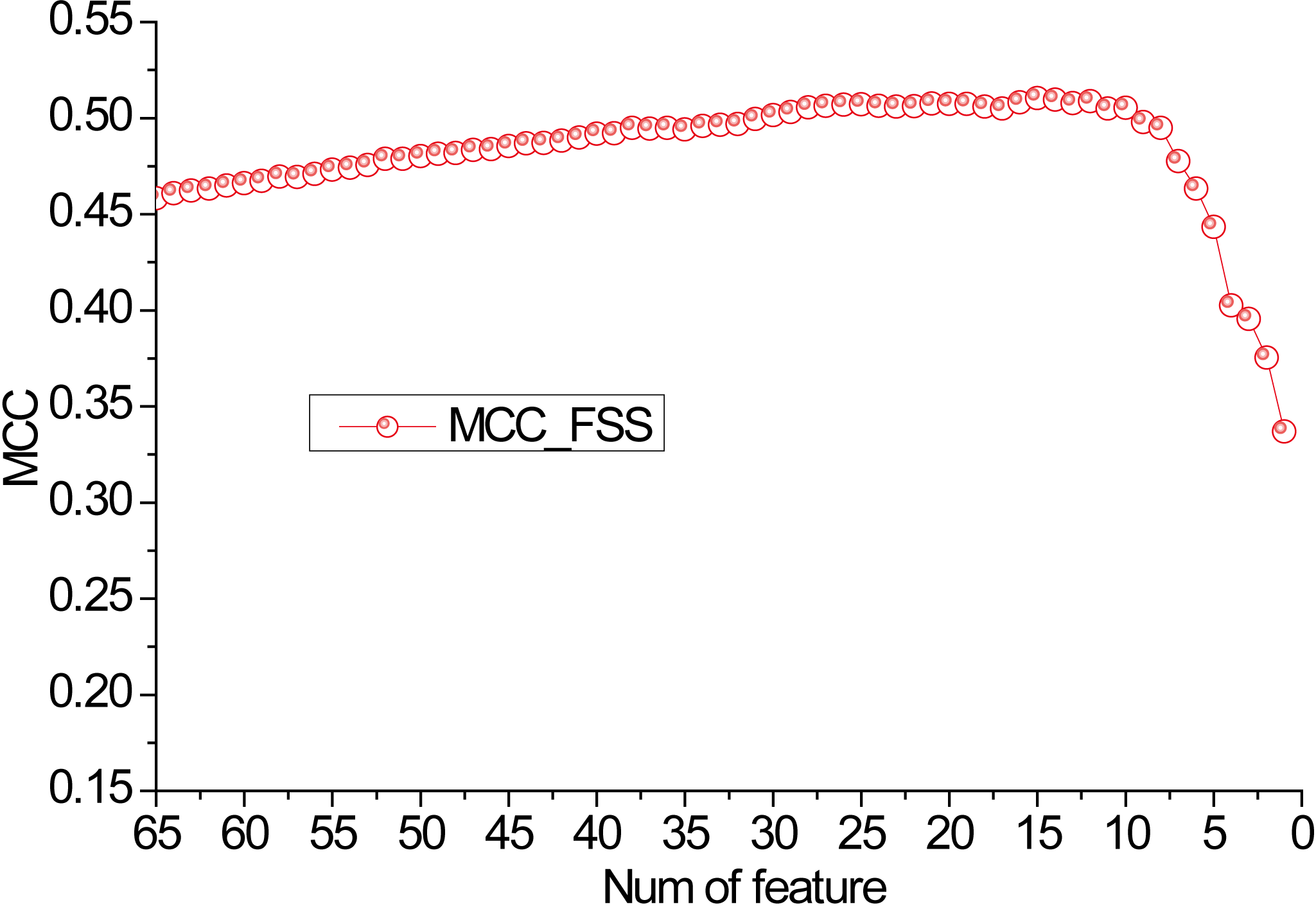

Supplement: Figure S1 — The feature selection curve in stepwise feature selection describes the performance change (in terms of MCC) of gradual inclusion of individual features to the trained classifiers. MCC_FSS (feature selection stepwise, FSS) indicates the MCC change in this stepwise feature selection process. (TIF) [file pone.0043847.s001.tif]
