# Supplementary material for: FunSAV: Predicting the Functional Effect of Single Amino Acid Variants Using a Two-Stage Random Forest Model
Source: PLoS One. 2012 Aug 24;7(8):e43847. doi: 10.1371/journal.pone.0043847 (PMC3427247; doi:10.1371/journal.pone.0043847)
Supplement: Table S1 — All initial 1804 features used in this study. “OFC” indicate that such feature was selected as the 65 optimal feature candidates (OFCs), while “FINAL” indicates that such feature was selected as one of the 15 final optimal features. (DOC) [file pone.0043847.s002.doc]

**Table S1. All initial 1804 features used in this study.**

“OFC” indicate that such feature was selected as the 65 optimal feature candidates (OFCs), while “FINAL” indicates that such feature was selected as one of the 15 final optimal features.

| feature name | feature name | feature name | feature name |
| --- | --- | --- | --- |
| Pfam_feature | tango_beta.V14 | uniprot_TRANSMEM_seq.V12 | uniprot_NP_BIND_seq.V10 |
| tango_turn.V2 | tango_beta.V15 | uniprot_TRANSMEM_seq.V13 | uniprot_NP_BIND_seq.V11 |
| tango_turn.V3 | tango_beta.V16 | uniprot_TRANSMEM_seq.V14 | uniprot_NP_BIND_seq.V12 |
| tango_turn.V4 | tango_Aggregation.V2 | uniprot_TRANSMEM_seq.V15 | uniprot_NP_BIND_seq.V13 |
| tango_turn.V5 | tango_Aggregation.V3 | uniprot_TRANSMEM_seq.V16 | uniprot_NP_BIND_seq.V14 |
| tango_turn.V6 | tango_Aggregation.V4 | uniprot_ZN_FING_seq.V2 | uniprot_NP_BIND_seq.V15 |
| tango_turn.V7 | tango_Aggregation.V5 | uniprot_ZN_FING_seq.V3 | uniprot_NP_BIND_seq.V16 |
| tango_turn.V8 | tango_Aggregation.V6 | uniprot_ZN_FING_seq.V4 | uniprot_DNA_BIND_seq.V2 |
| tango_turn.V9 | tango_Aggregation.V7 | uniprot_ZN_FING_seq.V5 | uniprot_DNA_BIND_seq.V3 |
| tango_turn.V10 | tango_Aggregation.V8 | uniprot_ZN_FING_seq.V6 | uniprot_DNA_BIND_seq.V4 |
| tango_turn.V11 | tango_Aggregation.V9 | uniprot_ZN_FING_seq.V7 | uniprot_DNA_BIND_seq.V5 |
| tango_turn.V12 | tango_Aggregation.V10 | uniprot_ZN_FING_seq.V8 | uniprot_DNA_BIND_seq.V6 |
| tango_turn.V13 | tango_Aggregation.V11 | uniprot_ZN_FING_seq.V9 | uniprot_DNA_BIND_seq.V7 |
| tango_turn.V14 | tango_Aggregation.V12 | uniprot_ZN_FING_seq.V10 | uniprot_DNA_BIND_seq.V8 |
| tango_turn.V15 | tango_Aggregation.V13 | uniprot_ZN_FING_seq.V11 | uniprot_DNA_BIND_seq.V9 |
| tango_turn.V16 | tango_Aggregation.V14 | uniprot_ZN_FING_seq.V12 | uniprot_DNA_BIND_seq.V10 |
| tango_helix.V2 | tango_Aggregation.V15 | uniprot_ZN_FING_seq.V13 | uniprot_DNA_BIND_seq.V11 |
| tango_helix.V3 | tango_Aggregation.V16 | uniprot_ZN_FING_seq.V14 | uniprot_DNA_BIND_seq.V12 |
| tango_helix.V4 | uniprot_DISULFID_seq.V2 | uniprot_ZN_FING_seq.V15 | uniprot_DNA_BIND_seq.V13 |
| tango_helix.V5 | uniprot_DISULFID_seq.V3 | uniprot_ZN_FING_seq.V16 | uniprot_DNA_BIND_seq.V14 |
| tango_helix.V6 | uniprot_DISULFID_seq.V4 | uniprot_MOTIF_seq.V2 | uniprot_DNA_BIND_seq.V15 |
| tango_helix.V7 | uniprot_DISULFID_seq.V5 | uniprot_MOTIF_seq.V3 | uniprot_DNA_BIND_seq.V16 |
| tango_helix.V8 | uniprot_DISULFID_seq.V6 | uniprot_MOTIF_seq.V4 | uniprot_METAL_seq.V2 |
| tango_helix.V9 | uniprot_DISULFID_seq.V7 | uniprot_MOTIF_seq.V5 | uniprot_METAL_seq.V3 |
| tango_helix.V10 | uniprot_DISULFID_seq.V8 | uniprot_MOTIF_seq.V6 | uniprot_METAL_seq.V4 |
| tango_helix.V11 | uniprot_DISULFID_seq.V9 | uniprot_MOTIF_seq.V7 | uniprot_METAL_seq.V5 |
| tango_helix.V12 | uniprot_DISULFID_seq.V10 | uniprot_MOTIF_seq.V8 | uniprot_METAL_seq.V6 |
| tango_helix.V13 | uniprot_DISULFID_seq.V11 | uniprot_MOTIF_seq.V9 | uniprot_METAL_seq.V7 |
| tango_helix.V14 | uniprot_DISULFID_seq.V12 | uniprot_MOTIF_seq.V10 | uniprot_METAL_seq.V8 |
| tango_helix.V15 | uniprot_DISULFID_seq.V13 | uniprot_MOTIF_seq.V11 | uniprot_METAL_seq.V9 |
| tango_helix.V16 | uniprot_DISULFID_seq.V14 | uniprot_MOTIF_seq.V12 | uniprot_METAL_seq.V10 |
| tango_beta.V2 | uniprot_DISULFID_seq.V15 | uniprot_MOTIF_seq.V13 | uniprot_METAL_seq.V11 |
| tango_beta.V3 | uniprot_DISULFID_seq.V16 | uniprot_MOTIF_seq.V14 | uniprot_METAL_seq.V12 |
| tango_beta.V4 | uniprot_TRANSMEM_seq.V2 | uniprot_MOTIF_seq.V15 | uniprot_METAL_seq.V13 |
| tango_beta.V5 | uniprot_TRANSMEM_seq.V3 | uniprot_MOTIF_seq.V16 | uniprot_METAL_seq.V14 |
| tango_beta.V6 | uniprot_TRANSMEM_seq.V4 | uniprot_NP_BIND_seq.V2 | uniprot_METAL_seq.V15 |
| tango_beta.V7 | uniprot_TRANSMEM_seq.V5 | uniprot_NP_BIND_seq.V3 | uniprot_METAL_seq.V16 |
| tango_beta.V8 | uniprot_TRANSMEM_seq.V6 | uniprot_NP_BIND_seq.V4 | uniprot_CROSSLNK_seq.V2 |
| tango_beta.V9 | uniprot_TRANSMEM_seq.V7 | uniprot_NP_BIND_seq.V5 | uniprot_CROSSLNK_seq.V3 |
| tango_beta.V10 | uniprot_TRANSMEM_seq.V8 | uniprot_NP_BIND_seq.V6 | uniprot_CROSSLNK_seq.V4 |
| tango_beta.V11 | uniprot_TRANSMEM_seq.V9 | uniprot_NP_BIND_seq.V7 | uniprot_CROSSLNK_seq.V5 |
| tango_beta.V12 | uniprot_TRANSMEM_seq.V10 | uniprot_NP_BIND_seq.V8 | uniprot_CROSSLNK_seq.V6 |
| tango_beta.V13 | uniprot_TRANSMEM_seq.V11 | uniprot_NP_BIND_seq.V9 | uniprot_CROSSLNK_seq.V7 |

| feature name | feature name | feature name | feature name |
| --- | --- | --- | --- |
| uniprot_CROSSLNK_seq.V8 | uniprot_BINDING_seq.V8 | uniprot_CA_BIND_seq.V8 | uniprot_DNA_BIND_3d.V8 |
| uniprot_CROSSLNK_seq.V9 | uniprot_BINDING_seq.V9 | uniprot_CA_BIND_seq.V9 | uniprot_DNA_BIND_3d.V9 |
| uniprot_CROSSLNK_seq.V10 | uniprot_BINDING_seq.V10 | uniprot_CA_BIND_seq.V10 | uniprot_DNA_BIND_3d.V10 |
| uniprot_CROSSLNK_seq.V11 | uniprot_BINDING_seq.V11 | uniprot_CA_BIND_seq.V11 | uniprot_DNA_BIND_3d.V11 |
| uniprot_CROSSLNK_seq.V12 | uniprot_BINDING_seq.V12 | uniprot_CA_BIND_seq.V12 | uniprot_DNA_BIND_3d.V12 |
| uniprot_CROSSLNK_seq.V13 | uniprot_BINDING_seq.V13 | uniprot_CA_BIND_seq.V13 | uniprot_DNA_BIND_3d.V13 |
| uniprot_CROSSLNK_seq.V14 | uniprot_BINDING_seq.V14 | uniprot_CA_BIND_seq.V14 | uniprot_DNA_BIND_3d.V14 |
| uniprot_CROSSLNK_seq.V15 | uniprot_BINDING_seq.V15 | uniprot_CA_BIND_seq.V15 | uniprot_DNA_BIND_3d.V15 |
| uniprot_CROSSLNK_seq.V16 | uniprot_BINDING_seq.V16 | uniprot_CA_BIND_seq.V16 | uniprot_DNA_BIND_3d.V16 |
| uniprot_LIPID_seq.V2 | uniprot_MOD_RES_seq.V2 | uniprot_NP_BIND_3d.V2 | uniprot_MOTIF_3d.V2 |
| uniprot_LIPID_seq.V3 | uniprot_MOD_RES_seq.V3 | uniprot_NP_BIND_3d.V3 | uniprot_MOTIF_3d.V3 |
| uniprot_LIPID_seq.V4 | uniprot_MOD_RES_seq.V4 | uniprot_NP_BIND_3d.V4 | uniprot_MOTIF_3d.V4 |
| uniprot_LIPID_seq.V5 | uniprot_MOD_RES_seq.V5 | uniprot_NP_BIND_3d.V5 | uniprot_MOTIF_3d.V5 |
| uniprot_LIPID_seq.V6 | uniprot_MOD_RES_seq.V6 | uniprot_NP_BIND_3d.V6 | uniprot_MOTIF_3d.V6 |
| uniprot_LIPID_seq.V7 | uniprot_MOD_RES_seq.V7 | uniprot_NP_BIND_3d.V7 | uniprot_MOTIF_3d.V7 |
| uniprot_LIPID_seq.V8 | uniprot_MOD_RES_seq.V8 | uniprot_NP_BIND_3d.V8 | uniprot_MOTIF_3d.V8 |
| uniprot_LIPID_seq.V9 | uniprot_MOD_RES_seq.V9 | uniprot_NP_BIND_3d.V9 | uniprot_MOTIF_3d.V9 |
| uniprot_LIPID_seq.V10 | uniprot_MOD_RES_seq.V10 | uniprot_NP_BIND_3d.V10 | uniprot_MOTIF_3d.V10 |
| uniprot_LIPID_seq.V11 | uniprot_MOD_RES_seq.V11 | uniprot_NP_BIND_3d.V11 | uniprot_MOTIF_3d.V11 |
| uniprot_LIPID_seq.V12 | uniprot_MOD_RES_seq.V12 | uniprot_NP_BIND_3d.V12 | uniprot_MOTIF_3d.V12 |
| uniprot_LIPID_seq.V13 | uniprot_MOD_RES_seq.V13 | uniprot_NP_BIND_3d.V13 | uniprot_MOTIF_3d.V13 |
| uniprot_LIPID_seq.V14 | uniprot_MOD_RES_seq.V14 | uniprot_NP_BIND_3d.V14 | uniprot_MOTIF_3d.V14 |
| uniprot_LIPID_seq.V15 | uniprot_MOD_RES_seq.V15 | uniprot_NP_BIND_3d.V15 | uniprot_MOTIF_3d.V15 |
| uniprot_LIPID_seq.V16 | uniprot_MOD_RES_seq.V16 | uniprot_NP_BIND_3d.V16 | uniprot_MOTIF_3d.V16 |
| uniprot_CARBOHYD_seq.V2 | uniprot_ACT_SITE_seq.V2 | uniprot_LIPID_3d.V2 | uniprot_MOD_RES_3d.V2 |
| uniprot_CARBOHYD_seq.V3 | uniprot_ACT_SITE_seq.V3 | uniprot_LIPID_3d.V3 | uniprot_MOD_RES_3d.V3 |
| uniprot_CARBOHYD_seq.V4 | uniprot_ACT_SITE_seq.V4 | uniprot_LIPID_3d.V4 | uniprot_MOD_RES_3d.V4 |
| uniprot_CARBOHYD_seq.V5 | uniprot_ACT_SITE_seq.V5 | uniprot_LIPID_3d.V5 | uniprot_MOD_RES_3d.V5 |
| uniprot_CARBOHYD_seq.V6 | uniprot_ACT_SITE_seq.V6 | uniprot_LIPID_3d.V6 | uniprot_MOD_RES_3d.V6 |
| uniprot_CARBOHYD_seq.V7 | uniprot_ACT_SITE_seq.V7 | uniprot_LIPID_3d.V7 | uniprot_MOD_RES_3d.V7 |
| uniprot_CARBOHYD_seq.V8 | uniprot_ACT_SITE_seq.V8 | uniprot_LIPID_3d.V8 | uniprot_MOD_RES_3d.V8 |
| uniprot_CARBOHYD_seq.V9 | uniprot_ACT_SITE_seq.V9 | uniprot_LIPID_3d.V9 | uniprot_MOD_RES_3d.V9 |
| uniprot_CARBOHYD_seq.V10 | uniprot_ACT_SITE_seq.V10 | uniprot_LIPID_3d.V10 | uniprot_MOD_RES_3d.V10 |
| uniprot_CARBOHYD_seq.V11 | uniprot_ACT_SITE_seq.V11 | uniprot_LIPID_3d.V11 | uniprot_MOD_RES_3d.V11 |
| uniprot_CARBOHYD_seq.V12 | uniprot_ACT_SITE_seq.V12 | uniprot_LIPID_3d.V12 | uniprot_MOD_RES_3d.V12 |
| uniprot_CARBOHYD_seq.V13 | uniprot_ACT_SITE_seq.V13 | uniprot_LIPID_3d.V13 | uniprot_MOD_RES_3d.V13 |
| uniprot_CARBOHYD_seq.V14 | uniprot_ACT_SITE_seq.V14 | uniprot_LIPID_3d.V14 | uniprot_MOD_RES_3d.V14 |
| uniprot_CARBOHYD_seq.V15 | uniprot_ACT_SITE_seq.V15 | uniprot_LIPID_3d.V15 | uniprot_MOD_RES_3d.V15 |
| uniprot_CARBOHYD_seq.V16 | uniprot_ACT_SITE_seq.V16 | uniprot_LIPID_3d.V16 | uniprot_MOD_RES_3d.V16 |
| uniprot_BINDING_seq.V2 | uniprot_CA_BIND_seq.V2 | uniprot_DNA_BIND_3d.V2 | uniprot_METAL_3d.V2 |
| uniprot_BINDING_seq.V3 | uniprot_CA_BIND_seq.V3 | uniprot_DNA_BIND_3d.V3 | uniprot_METAL_3d.V3 |
| uniprot_BINDING_seq.V4 | uniprot_CA_BIND_seq.V4 | uniprot_DNA_BIND_3d.V4 | uniprot_METAL_3d.V4 |
| uniprot_BINDING_seq.V5 | uniprot_CA_BIND_seq.V5 | uniprot_DNA_BIND_3d.V5 | uniprot_METAL_3d.V5 |
| uniprot_BINDING_seq.V6 | uniprot_CA_BIND_seq.V6 | uniprot_DNA_BIND_3d.V6 | uniprot_METAL_3d.V6 |
| uniprot_BINDING_seq.V7 | uniprot_CA_BIND_seq.V7 | uniprot_DNA_BIND_3d.V7 | uniprot_METAL_3d.V7 |

| feature name | feature name | feature name | feature name |
| --- | --- | --- | --- |
| uniprot_METAL_3d.V8 | uniprot_CROSSLNK_3d.V7 | uniprot_NP_BIND_3d.V6.1 | aa_15.V5 |
| uniprot_METAL_3d.V9 | uniprot_CROSSLNK_3d.V8 | uniprot_NP_BIND_3d.V7.1 | aa_15.V6 |
| uniprot_METAL_3d.V10 | uniprot_CROSSLNK_3d.V9 | uniprot_NP_BIND_3d.V8.1 | aa_15.V7 |
| uniprot_METAL_3d.V11 | uniprot_CROSSLNK_3d.V10 | uniprot_NP_BIND_3d.V9.1 | aa_15.V8 |
| uniprot_METAL_3d.V12 | uniprot_CROSSLNK_3d.V11 | uniprot_NP_BIND_3d.V10.1 | aa_15.V9 |
| uniprot_METAL_3d.V13 | uniprot_CROSSLNK_3d.V12 | uniprot_NP_BIND_3d.V11.1 | aa_15.V10 |
| uniprot_METAL_3d.V14 | uniprot_CROSSLNK_3d.V13 | uniprot_NP_BIND_3d.V12.1 | aa_15.V11 |
| uniprot_METAL_3d.V15 | uniprot_CROSSLNK_3d.V14 | uniprot_NP_BIND_3d.V13.1 | aa_15.V12 |
| uniprot_METAL_3d.V16 | uniprot_CROSSLNK_3d.V15 | uniprot_NP_BIND_3d.V14.1 | aa_15.V13 |
| uniprot_TRANSMEM_3d.V2 | uniprot_CROSSLNK_3d.V16 | uniprot_NP_BIND_3d.V15.1 | aa_15.V14 |
| uniprot_TRANSMEM_3d.V3 | uniprot_CA_BIND_3d.V2 | uniprot_NP_BIND_3d.V16.1 | aa_15.V15 |
| uniprot_TRANSMEM_3d.V4 | uniprot_CA_BIND_3d.V3 | uniprot_CARBOHYD_3d.V2 | aa_15.V16 |
| uniprot_TRANSMEM_3d.V5 | uniprot_CA_BIND_3d.V4 | uniprot_CARBOHYD_3d.V3 | dis_3d_0.V2 |
| uniprot_TRANSMEM_3d.V6 | uniprot_CA_BIND_3d.V5 | uniprot_CARBOHYD_3d.V4 | dis_3d_0.V3 |
| uniprot_TRANSMEM_3d.V7 | uniprot_CA_BIND_3d.V6 | uniprot_CARBOHYD_3d.V5 | dis_3d_0.V4 |
| uniprot_TRANSMEM_3d.V8 | uniprot_CA_BIND_3d.V7 | uniprot_CARBOHYD_3d.V6 | dis_3d_0.V5 |
| uniprot_TRANSMEM_3d.V9 | uniprot_CA_BIND_3d.V8 | uniprot_CARBOHYD_3d.V7 | dis_3d_0.V6 |
| uniprot_TRANSMEM_3d.V10 | uniprot_CA_BIND_3d.V9 | uniprot_CARBOHYD_3d.V8 | dis_3d_0.V7 |
| uniprot_TRANSMEM_3d.V11 | uniprot_CA_BIND_3d.V10 | uniprot_CARBOHYD_3d.V9 | dis_3d_0.V8 |
| uniprot_TRANSMEM_3d.V12 | uniprot_CA_BIND_3d.V11 | uniprot_CARBOHYD_3d.V10 | dis_3d_0.V9 |
| uniprot_TRANSMEM_3d.V13 | uniprot_CA_BIND_3d.V12 | uniprot_CARBOHYD_3d.V11 | dis_3d_0.V10 |
| uniprot_TRANSMEM_3d.V14 | uniprot_CA_BIND_3d.V13 | uniprot_CARBOHYD_3d.V12 | dis_3d_0.V11 |
| uniprot_TRANSMEM_3d.V15 | uniprot_CA_BIND_3d.V14 | uniprot_CARBOHYD_3d.V13 | dis_3d_0.V12 |
| uniprot_TRANSMEM_3d.V16 | uniprot_CA_BIND_3d.V15 | uniprot_CARBOHYD_3d.V14 | dis_3d_0.V13 |
| uniprot_ZN_FING_3d.V2 | uniprot_CA_BIND_3d.V16 | uniprot_CARBOHYD_3d.V15 | dis_3d_0.V14 |
| uniprot_ZN_FING_3d.V3 | uniprot_DISULFID_3d.V2 | uniprot_CARBOHYD_3d.V16 | dis_3d_0.V15 |
| uniprot_ZN_FING_3d.V4 | uniprot_DISULFID_3d.V3 | uniprot_act_site_3d.V2 | dis_3d_0.V16 |
| uniprot_ZN_FING_3d.V5 | uniprot_DISULFID_3d.V4 | uniprot_act_site_3d.V3 | mut_aa.V2 |
| uniprot_ZN_FING_3d.V6 | uniprot_DISULFID_3d.V5 | uniprot_act_site_3d.V4 | mut_aa.V3 |
| uniprot_ZN_FING_3d.V7 | uniprot_DISULFID_3d.V6 | uniprot_act_site_3d.V5 | mw_change_v1_w.V2; OFC FINAL |
| uniprot_ZN_FING_3d.V8 | uniprot_DISULFID_3d.V7 | uniprot_act_site_3d.V6 | conserve_score_win.V2 |
| uniprot_ZN_FING_3d.V9 | uniprot_DISULFID_3d.V8 | uniprot_act_site_3d.V7 | conserve_score_win.V3 |
| uniprot_ZN_FING_3d.V10 | uniprot_DISULFID_3d.V9 | uniprot_act_site_3d.V8 | conserve_score_win.V4 |
| uniprot_ZN_FING_3d.V11 | uniprot_DISULFID_3d.V10 | uniprot_act_site_3d.V9 | conserve_score_win.V5 |
| uniprot_ZN_FING_3d.V12 | uniprot_DISULFID_3d.V11 | uniprot_act_site_3d.V10 | conserve_score_win.V6 |
| uniprot_ZN_FING_3d.V13 | uniprot_DISULFID_3d.V12 | uniprot_act_site_3d.V11 | conserve_score_win.V7 |
| uniprot_ZN_FING_3d.V14 | uniprot_DISULFID_3d.V13 | uniprot_act_site_3d.V12 | conserve_score_win.V8 |
| uniprot_ZN_FING_3d.V15 | uniprot_DISULFID_3d.V14 | uniprot_act_site_3d.V13 | conserve_score_win.V9; OFC FINAL |
| uniprot_ZN_FING_3d.V16 | uniprot_DISULFID_3d.V15 | uniprot_act_site_3d.V14 | conserve_score_win.V10 |
| uniprot_CROSSLNK_3d.V2 | uniprot_DISULFID_3d.V16 | uniprot_act_site_3d.V15 | conserve_score_win.V11 |
| uniprot_CROSSLNK_3d.V3 | uniprot_NP_BIND_3d.V2.1 | uniprot_act_site_3d.V16 | conserve_score_win.V12 |
| uniprot_CROSSLNK_3d.V4 | uniprot_NP_BIND_3d.V3.1 | aa_15.V2 | conserve_score_win.V13 |
| uniprot_CROSSLNK_3d.V5 | uniprot_NP_BIND_3d.V4.1 | aa_15.V3 | conserve_score_win.V14 |
| uniprot_CROSSLNK_3d.V6 | uniprot_NP_BIND_3d.V5.1 | aa_15.V4 | conserve_score_win.V15 |

| feature name | feature name | feature name | feature name |
| --- | --- | --- | --- |
| conserve_score_win.V16 | disopred.V46 | psipred_encode.V46 | pssm.V31 |
| disopred.V2 | psipred_encode.V2 | psipred_encode.V47 | pssm.V32 |
| disopred.V3 | psipred_encode.V3 | psipred_encode.V48 | pssm.V33 |
| disopred.V4 | psipred_encode.V4 | psipred_encode.V49 | pssm.V34 |
| disopred.V5 | psipred_encode.V5 | psipred_encode.V50 | pssm.V35 |
| disopred.V6 | psipred_encode.V6 | psipred_encode.V51 | pssm.V36 |
| disopred.V7 | psipred_encode.V7 | psipred_encode.V52 | pssm.V37 |
| disopred.V8 | psipred_encode.V8 | psipred_encode.V53 | pssm.V38 |
| disopred.V9 | psipred_encode.V9 | psipred_encode.V54 | pssm.V39 |
| disopred.V10 | psipred_encode.V10 | psipred_encode.V55 | pssm.V40 |
| disopred.V11 | psipred_encode.V11 | psipred_encode.V56 | pssm.V41 |
| disopred.V12 | psipred_encode.V12 | psipred_encode.V57 | pssm.V42 |
| disopred.V13 | psipred_encode.V13 | psipred_encode.V58 | pssm.V43 |
| disopred.V14 | psipred_encode.V14 | psipred_encode.V59 | pssm.V44 |
| disopred.V15 | psipred_encode.V15 | psipred_encode.V60 | pssm.V45 |
| disopred.V16 | psipred_encode.V16 | psipred_encode.V61 | pssm.V46 |
| disopred.V17 | psipred_encode.V17 | pssm.V2 | pssm.V47 |
| disopred.V18; OFC | psipred_encode.V18 | pssm.V3 | pssm.V48 |
| disopred.V19 | psipred_encode.V19 | pssm.V4 | pssm.V49 |
| disopred.V20 | psipred_encode.V20 | pssm.V5 | pssm.V50 |
| disopred.V21 | psipred_encode.V21 | pssm.V6 | pssm.V51 |
| disopred.V22 | psipred_encode.V22 | pssm.V7 | pssm.V52 |
| disopred.V23 | psipred_encode.V23 | pssm.V8 | pssm.V53 |
| disopred.V24 | psipred_encode.V24 | pssm.V9 | pssm.V54 |
| disopred.V25 | psipred_encode.V25 | pssm.V10 | pssm.V55 |
| disopred.V26 | psipred_encode.V26 | pssm.V11 | pssm.V56 |
| disopred.V27 | psipred_encode.V27 | pssm.V12 | pssm.V57 |
| disopred.V28 | psipred_encode.V28 | pssm.V13 | pssm.V58 |
| disopred.V29 | psipred_encode.V29 | pssm.V14 | pssm.V59 |
| disopred.V30 | psipred_encode.V30 | pssm.V15 | pssm.V60 |
| disopred.V31 | psipred_encode.V31 | pssm.V16 | pssm.V61 |
| disopred.V32 | psipred_encode.V32 | pssm.V17 | pssm.V62 |
| disopred.V33 | psipred_encode.V33 | pssm.V18 | pssm.V63 |
| disopred.V34 | psipred_encode.V34 | pssm.V19 | pssm.V64 |
| disopred.V35 | psipred_encode.V35 | pssm.V20 | pssm.V65 |
| disopred.V36 | psipred_encode.V36 | pssm.V21 | pssm.V66 |
| disopred.V37 | psipred_encode.V37 | pssm.V22 | pssm.V67 |
| disopred.V38 | psipred_encode.V38 | pssm.V23 | pssm.V68 |
| disopred.V39 | psipred_encode.V39 | pssm.V24 | pssm.V69 |
| disopred.V40 | psipred_encode.V40 | pssm.V25 | pssm.V70 |
| disopred.V41 | psipred_encode.V41 | pssm.V26 | pssm.V71 |
| disopred.V42 | psipred_encode.V42 | pssm.V27 | pssm.V72 |
| disopred.V43 | psipred_encode.V43 | pssm.V28 | pssm.V73 |
| disopred.V44 | psipred_encode.V44 | pssm.V29 | pssm.V74 |
| disopred.V45 | psipred_encode.V45 | pssm.V30 | pssm.V75 |

| feature name | feature name | feature name | feature name |
| --- | --- | --- | --- |
| pssm.V76 | pssm.V121 | pssm.V166 | pssm.V211 |
| pssm.V77 | pssm.V122 | pssm.V167 | pssm.V212 |
| pssm.V78 | pssm.V123 | pssm.V168 | pssm.V213 |
| pssm.V79 | pssm.V124 | pssm.V169 | pssm.V214 |
| pssm.V80 | pssm.V125 | pssm.V170 | pssm.V215 |
| pssm.V81 | pssm.V126 | pssm.V171 | pssm.V216 |
| pssm.V82 | pssm.V127 | pssm.V172 | pssm.V217 |
| pssm.V83 | pssm.V128 | pssm.V173 | pssm.V218 |
| pssm.V84 | pssm.V129 | pssm.V174 | pssm.V219 |
| pssm.V85 | pssm.V130 | pssm.V175 | pssm.V220 |
| pssm.V86 | pssm.V131 | pssm.V176 | pssm.V221 |
| pssm.V87 | pssm.V132 | pssm.V177 | pssm.V222 |
| pssm.V88 | pssm.V133 | pssm.V178 | pssm.V223 |
| pssm.V89 | pssm.V134 | pssm.V179 | pssm.V224 |
| pssm.V90 | pssm.V135 | pssm.V180 | pssm.V225 |
| pssm.V91 | pssm.V136 | pssm.V181 | pssm.V226 |
| pssm.V92 | pssm.V137 | pssm.V182 | pssm.V227 |
| pssm.V93 | pssm.V138 | pssm.V183 | pssm.V228 |
| pssm.V94 | pssm.V139 | pssm.V184 | pssm.V229 |
| pssm.V95 | pssm.V140 | pssm.V185 | pssm.V230 |
| pssm.V96 | pssm.V141 | pssm.V186 | pssm.V231 |
| pssm.V97 | pssm.V142; OFC | pssm.V187 | pssm.V232 |
| pssm.V98 | pssm.V143; OFC | pssm.V188 | pssm.V233 |
| pssm.V99 | pssm.V144; OFC | pssm.V189 | pssm.V234 |
| pssm.V100 | pssm.V145; OFC | pssm.V190 | pssm.V235 |
| pssm.V101 | pssm.V146 | pssm.V191 | pssm.V236 |
| pssm.V102 | pssm.V147; OFC | pssm.V192 | pssm.V237 |
| pssm.V103 | pssm.V148; OFC | pssm.V193 | pssm.V238 |
| pssm.V104 | pssm.V149 | pssm.V194 | pssm.V239 |
| pssm.V105 | pssm.V150; OFC | pssm.V195 | pssm.V240 |
| pssm.V106 | pssm.V151 | pssm.V196 | pssm.V241 |
| pssm.V107 | pssm.V152 | pssm.V197 | pssm.V242 |
| pssm.V108 | pssm.V153; OFC | pssm.V198 | pssm.V243 |
| pssm.V109 | pssm.V154; OFC | pssm.V199 | pssm.V244 |
| pssm.V110 | pssm.V155 | pssm.V200 | pssm.V245 |
| pssm.V111 | pssm.V156; OFC | pssm.V201 | pssm.V246 |
| pssm.V112 | pssm.V157; OFC | pssm.V202 | pssm.V247 |
| pssm.V113 | pssm.V158; OFC | pssm.V203 | pssm.V248 |
| pssm.V114 | pssm.V159 | pssm.V204 | pssm.V249 |
| pssm.V115 | pssm.V160 | pssm.V205 | pssm.V250 |
| pssm.V116 | pssm.V161; OFC; FINAL | pssm.V206 | pssm.V251 |
| pssm.V117 | pssm.V162 | pssm.V207 | pssm.V252 |
| pssm.V118 | pssm.V163 | pssm.V208 | pssm.V253 |
| pssm.V119 | pssm.V164 | pssm.V209 | pssm.V254 |
| pssm.V120 | pssm.V165 | pssm.V210 | pssm.V255 |

| feature name | feature name | feature name | feature name |
| --- | --- | --- | --- |
| pssm.V256 | pssm.V301 | dssp_2nd_N_H_O.V31 | dssp_acc.V16 |
| pssm.V257 | sspro_code.V2 | dssp_2nd_O_H_N.V2 | dssp_alpha.V2 |
| pssm.V258 | sspro_code.V3 | dssp_2nd_O_H_N.V3 | dssp_alpha.V3 |
| pssm.V259 | sspro_code.V4 | dssp_2nd_O_H_N.V4 | dssp_alpha.V4 |
| pssm.V260 | sspro_code.V5 | dssp_2nd_O_H_N.V5 | dssp_alpha.V5 |
| pssm.V261 | sspro_code.V6 | dssp_2nd_O_H_N.V6 | dssp_alpha.V6 |
| pssm.V262 | sspro_code.V7 | dssp_2nd_O_H_N.V7 | dssp_alpha.V7 |
| pssm.V263 | sspro_code.V8 | dssp_2nd_O_H_N.V8 | dssp_alpha.V8 |
| pssm.V264 | sspro_code.V9; OFC FINAL | dssp_2nd_O_H_N.V9 | dssp_alpha.V9 |
| pssm.V265 | sspro_code.V10 | dssp_2nd_O_H_N.V10 | dssp_alpha.V10 |
| pssm.V266 | sspro_code.V11 | dssp_2nd_O_H_N.V11 | dssp_alpha.V11 |
| pssm.V267 | sspro_code.V12 | dssp_2nd_O_H_N.V12 | dssp_alpha.V12 |
| pssm.V268 | sspro_code.V13 | dssp_2nd_O_H_N.V13 | dssp_alpha.V13 |
| pssm.V269 | sspro_code.V14 | dssp_2nd_O_H_N.V14 | dssp_alpha.V14 |
| pssm.V270 | sspro_code.V15 | dssp_2nd_O_H_N.V15 | dssp_alpha.V15 |
| pssm.V271 | sspro_code.V16 | dssp_2nd_O_H_N.V16 | dssp_alpha.V16 |
| pssm.V272 | dssp_2nd_N_H_O.V2 | dssp_2nd_O_H_N.V17 | dssp_kappa.V2 |
| pssm.V273 | dssp_2nd_N_H_O.V3 | dssp_2nd_O_H_N.V18 | dssp_kappa.V3 |
| pssm.V274 | dssp_2nd_N_H_O.V4 | dssp_2nd_O_H_N.V19 | dssp_kappa.V4 |
| pssm.V275 | dssp_2nd_N_H_O.V5 | dssp_2nd_O_H_N.V20 | dssp_kappa.V5 |
| pssm.V276 | dssp_2nd_N_H_O.V6 | dssp_2nd_O_H_N.V21 | dssp_kappa.V6 |
| pssm.V277 | dssp_2nd_N_H_O.V7 | dssp_2nd_O_H_N.V22 | dssp_kappa.V7 |
| pssm.V278 | dssp_2nd_N_H_O.V8 | dssp_2nd_O_H_N.V23 | dssp_kappa.V8 |
| pssm.V279 | dssp_2nd_N_H_O.V9 | dssp_2nd_O_H_N.V24 | dssp_kappa.V9 |
| pssm.V280 | dssp_2nd_N_H_O.V10 | dssp_2nd_O_H_N.V25 | dssp_kappa.V10 |
| pssm.V281 | dssp_2nd_N_H_O.V11 | dssp_2nd_O_H_N.V26 | dssp_kappa.V11 |
| pssm.V282 | dssp_2nd_N_H_O.V12 | dssp_2nd_O_H_N.V27 | dssp_kappa.V12 |
| pssm.V283 | dssp_2nd_N_H_O.V13 | dssp_2nd_O_H_N.V28 | dssp_kappa.V13 |
| pssm.V284 | dssp_2nd_N_H_O.V14 | dssp_2nd_O_H_N.V29 | dssp_kappa.V14 |
| pssm.V285 | dssp_2nd_N_H_O.V15 | dssp_2nd_O_H_N.V30 | dssp_kappa.V15 |
| pssm.V286 | dssp_2nd_N_H_O.V16 | dssp_2nd_O_H_N.V31 | dssp_kappa.V16 |
| pssm.V287 | dssp_2nd_N_H_O.V17 | dssp_acc.V2 | dssp_N_H_O.V2 |
| pssm.V288 | dssp_2nd_N_H_O.V18 | dssp_acc.V3 | dssp_N_H_O.V3 |
| pssm.V289 | dssp_2nd_N_H_O.V19 | dssp_acc.V4 | dssp_N_H_O.V4 |
| pssm.V290 | dssp_2nd_N_H_O.V20 | dssp_acc.V5 | dssp_N_H_O.V5 |
| pssm.V291 | dssp_2nd_N_H_O.V21 | dssp_acc.V6 | dssp_N_H_O.V6 |
| pssm.V292 | dssp_2nd_N_H_O.V22 | dssp_acc.V7 | dssp_N_H_O.V7 |
| pssm.V293 | dssp_2nd_N_H_O.V23 | dssp_acc.V8 | dssp_N_H_O.V8 |
| pssm.V294 | dssp_2nd_N_H_O.V24 | dssp_acc.V9; OFC | dssp_N_H_O.V9 |
| pssm.V295 | dssp_2nd_N_H_O.V25 | dssp_acc.V10 | dssp_N_H_O.V10 |
| pssm.V296 | dssp_2nd_N_H_O.V26 | dssp_acc.V11 | dssp_N_H_O.V11 |
| pssm.V297 | dssp_2nd_N_H_O.V27 | dssp_acc.V12 | dssp_N_H_O.V12 |
| pssm.V298 | dssp_2nd_N_H_O.V28 | dssp_acc.V13 | dssp_N_H_O.V13 |
| pssm.V299 | dssp_2nd_N_H_O.V29 | dssp_acc.V14 | dssp_N_H_O.V14 |
| pssm.V300 | dssp_2nd_N_H_O.V30 | dssp_acc.V15 | dssp_N_H_O.V15 |

| feature name | feature name | feature name | feature name |
| --- | --- | --- | --- |
| dssp_N_H_O.V16 | dssp_O_H_N.V31 | dssp_stru.V16 | dssp_y_ca.V16 |
| dssp_N_H_O.V17 | dssp_phi.V2 | dssp_tco.V2 | dssp_z_ca.V2 |
| dssp_N_H_O.V18 | dssp_phi.V3 | dssp_tco.V3 | dssp_z_ca.V3 |
| dssp_N_H_O.V19 | dssp_phi.V4 | dssp_tco.V4 | dssp_z_ca.V4 |
| dssp_N_H_O.V20 | dssp_phi.V5 | dssp_tco.V5 | dssp_z_ca.V5 |
| dssp_N_H_O.V21 | dssp_phi.V6 | dssp_tco.V6 | dssp_z_ca.V6 |
| dssp_N_H_O.V22 | dssp_phi.V7 | dssp_tco.V7 | dssp_z_ca.V7 |
| dssp_N_H_O.V23 | dssp_phi.V8 | dssp_tco.V8 | dssp_z_ca.V8 |
| dssp_N_H_O.V24 | dssp_phi.V9 | dssp_tco.V9 | dssp_z_ca.V9 |
| dssp_N_H_O.V25 | dssp_phi.V10 | dssp_tco.V10 | dssp_z_ca.V10 |
| dssp_N_H_O.V26 | dssp_phi.V11 | dssp_tco.V11 | dssp_z_ca.V11 |
| dssp_N_H_O.V27 | dssp_phi.V12 | dssp_tco.V12 | dssp_z_ca.V12 |
| dssp_N_H_O.V28 | dssp_phi.V13 | dssp_tco.V13 | dssp_z_ca.V13 |
| dssp_N_H_O.V29 | dssp_phi.V14 | dssp_tco.V14 | dssp_z_ca.V14 |
| dssp_N_H_O.V30 | dssp_phi.V15 | dssp_tco.V15 | dssp_z_ca.V15 |
| dssp_N_H_O.V31 | dssp_phi.V16 | dssp_tco.V16 | dssp_z_ca.V16 |
| dssp_O_H_N.V2 | dssp_psi.V2 | dssp_x_ca.V2 | exposure_b_factor.V2 |
| dssp_O_H_N.V3 | dssp_psi.V3 | dssp_x_ca.V3 | exposure_b_factor.V3 |
| dssp_O_H_N.V4 | dssp_psi.V4 | dssp_x_ca.V4 | exposure_b_factor.V4 |
| dssp_O_H_N.V5 | dssp_psi.V5 | dssp_x_ca.V5 | exposure_b_factor.V5 |
| dssp_O_H_N.V6 | dssp_psi.V6 | dssp_x_ca.V6 | exposure_b_factor.V6; OFC |
| dssp_O_H_N.V7 | dssp_psi.V7 | dssp_x_ca.V7 | exposure_b_factor.V7 |
| dssp_O_H_N.V8 | dssp_psi.V8 | dssp_x_ca.V8 | exposure_b_factor.V8; OFC FINAL |
| dssp_O_H_N.V9 | dssp_psi.V9 | dssp_x_ca.V9 | exposure_b_factor.V9; OFC |
| dssp_O_H_N.V10 | dssp_psi.V10 | dssp_x_ca.V10 | exposure_b_factor.V10; OFC |
| dssp_O_H_N.V11 | dssp_psi.V11 | dssp_x_ca.V11 | exposure_b_factor.V11 |
| dssp_O_H_N.V12 | dssp_psi.V12 | dssp_x_ca.V12 | exposure_b_factor.V12; OFC |
| dssp_O_H_N.V13 | dssp_psi.V13 | dssp_x_ca.V13 | exposure_b_factor.V13 |
| dssp_O_H_N.V14 | dssp_psi.V14 | dssp_x_ca.V14 | exposure_b_factor.V14 |
| dssp_O_H_N.V15 | dssp_psi.V15 | dssp_x_ca.V15 | exposure_b_factor.V15 |
| dssp_O_H_N.V16 | dssp_psi.V16 | dssp_x_ca.V16 | exposure_b_factor.V16 |
| dssp_O_H_N.V17 | dssp_stru.V2 | dssp_y_ca.V2 | exposure_CN.V2 |
| dssp_O_H_N.V18 | dssp_stru.V3 | dssp_y_ca.V3 | exposure_CN.V3 |
| dssp_O_H_N.V19 | dssp_stru.V4 | dssp_y_ca.V4 | exposure_CN.V4 |
| dssp_O_H_N.V20 | dssp_stru.V5 | dssp_y_ca.V5 | exposure_CN.V5 |
| dssp_O_H_N.V21 | dssp_stru.V6 | dssp_y_ca.V6 | exposure_CN.V6; OFC |
| dssp_O_H_N.V22 | dssp_stru.V7 | dssp_y_ca.V7 | exposure_CN.V7 |
| dssp_O_H_N.V23 | dssp_stru.V8 | dssp_y_ca.V8 | exposure_CN.V8; OFC |
| dssp_O_H_N.V24 | dssp_stru.V9 | dssp_y_ca.V9 | exposure_CN.V9; OFC |
| dssp_O_H_N.V25 | dssp_stru.V10 | dssp_y_ca.V10 | exposure_CN.V10; OFC FINAL |
| dssp_O_H_N.V26 | dssp_stru.V11 | dssp_y_ca.V11 | exposure_CN.V11 |
| dssp_O_H_N.V27 | dssp_stru.V12 | dssp_y_ca.V12 | exposure_CN.V12 |
| dssp_O_H_N.V28 | dssp_stru.V13 | dssp_y_ca.V13 | exposure_CN.V13 |
| dssp_O_H_N.V29 | dssp_stru.V14 | dssp_y_ca.V14 | exposure_CN.V14 |
| dssp_O_H_N.V30 | dssp_stru.V15 | dssp_y_ca.V15 | exposure_CN.V15 |

| feature name | feature name | feature name | feature name |
| --- | --- | --- | --- |
| exposure_CN.V16 | exposure_HSEBD.V16 | exposure_RDa.V16 | naccess_all_atom_rel.V16 |
| exposure_HSEAD.V2 | exposure_HSEBU.V2 | hbplus.V2 | naccess_all_polar_abs.V2 |
| exposure_HSEAD.V3 | exposure_HSEBU.V3 | hbplus.V3 | naccess_all_polar_abs.V3 |
| exposure_HSEAD.V4 | exposure_HSEBU.V4 | hbplus.V4 | naccess_all_polar_abs.V4 |
| exposure_HSEAD.V5 | exposure_HSEBU.V5 | hbplus.V5 | naccess_all_polar_abs.V5 |
| exposure_HSEAD.V6 | exposure_HSEBU.V6 | hbplus.V6 | naccess_all_polar_abs.V6 |
| exposure_HSEAD.V7 | exposure_HSEBU.V7 | hbplus.V7 | naccess_all_polar_abs.V7 |
| exposure_HSEAD.V8; OFC | exposure_HSEBU.V8 | hbplus.V8 | naccess_all_polar_abs.V8 |
| exposure_HSEAD.V9 | exposure_HSEBU.V9 | hbplus.V9 | naccess_all_polar_abs.V9; OFC |
| exposure_HSEAD.V10; OFC | exposure_HSEBU.V10; OFC FINAL | hbplus.V10 | naccess_all_polar_abs.V10 |
| exposure_HSEAD.V11 | exposure_HSEBU.V11 | hbplus.V11 | naccess_all_polar_abs.V11 |
| exposure_HSEAD.V12 | exposure_HSEBU.V12 | hbplus.V12 | naccess_all_polar_abs.V12 |
| exposure_HSEAD.V13 | exposure_HSEBU.V13 | hbplus.V13 | naccess_all_polar_abs.V13 |
| exposure_HSEAD.V14 | exposure_HSEBU.V14 | hbplus.V14 | naccess_all_polar_abs.V14 |
| exposure_HSEAD.V15 | exposure_HSEBU.V15 | hbplus.V15 | naccess_all_polar_abs.V15 |
| exposure_HSEAD.V16 | exposure_HSEBU.V16 | hbplus.V16 | naccess_all_polar_abs.V16 |
| exposure_HSEAU.V2 | exposure_RD.V2 | naccess_all_atom_abs.V2 | naccess_all_polar_rel.V2 |
| exposure_HSEAU.V3 | exposure_RD.V3 | naccess_all_atom_abs.V3 | naccess_all_polar_rel.V3 |
| exposure_HSEAU.V4 | exposure_RD.V4 | naccess_all_atom_abs.V4 | naccess_all_polar_rel.V4 |
| exposure_HSEAU.V5 | exposure_RD.V5 | naccess_all_atom_abs.V5 | naccess_all_polar_rel.V5 |
| exposure_HSEAU.V6 | exposure_RD.V6 | naccess_all_atom_abs.V6 | naccess_all_polar_rel.V6 |
| exposure_HSEAU.V7 | exposure_RD.V7 | naccess_all_atom_abs.V7 | naccess_all_polar_rel.V7 |
| exposure_HSEAU.V8 | exposure_RD.V8 | naccess_all_atom_abs.V8 | naccess_all_polar_rel.V8 |
| exposure_HSEAU.V9; OFC | exposure_RD.V9; OFC FINAL | naccess_all_atom_abs.V9 OFC | naccess_all_polar_rel.V9; OFC |
| exposure_HSEAU.V10 | exposure_RD.V10 | naccess_all_atom_abs.V10 | naccess_all_polar_rel.V10 |
| exposure_HSEAU.V11 | exposure_RD.V11 | naccess_all_atom_abs.V11 | naccess_all_polar_rel.V11 |
| exposure_HSEAU.V12 | exposure_RD.V12 | naccess_all_atom_abs.V12 | naccess_all_polar_rel.V12 |
| exposure_HSEAU.V13 | exposure_RD.V13 | naccess_all_atom_abs.V13 | naccess_all_polar_rel.V13 |
| exposure_HSEAU.V14 | exposure_RD.V14 | naccess_all_atom_abs.V14 | naccess_all_polar_rel.V14 |
| exposure_HSEAU.V15 | exposure_RD.V15 | naccess_all_atom_abs.V15 | naccess_all_polar_rel.V15 |
| exposure_HSEAU.V16 | exposure_RD.V16 | naccess_all_atom_abs.V16 | naccess_all_polar_rel.V16 |
| exposure_HSEBD.V2 | exposure_RDa.V2 | naccess_all_atom_rel.V2 | naccess_main_chain_abs.V2 |
| exposure_HSEBD.V3 | exposure_RDa.V3 | naccess_all_atom_rel.V3 | naccess_main_chain_abs.V3 |
| exposure_HSEBD.V4 | exposure_RDa.V4 | naccess_all_atom_rel.V4 | naccess_main_chain_abs.V4 |
| exposure_HSEBD.V5 | exposure_RDa.V5 | naccess_all_atom_rel.V5 | naccess_main_chain_abs.V5 |
| exposure_HSEBD.V6 | exposure_RDa.V6 | naccess_all_atom_rel.V6 | naccess_main_chain_abs.V6 |
| exposure_HSEBD.V7 | exposure_RDa.V7 | naccess_all_atom_rel.V7 | naccess_main_chain_abs.V7 |
| exposure_HSEBD.V8 | exposure_RDa.V8 | naccess_all_atom_rel.V8 | naccess_main_chain_abs.V8 |
| exposure_HSEBD.V9; OFC FINAL | exposure_RDa.V9; OFC | naccess_all_atom_rel.V9 OFC | naccess_main_chain_abs.V9 |
| exposure_HSEBD.V10 | exposure_RDa.V10 | naccess_all_atom_rel.V10 | naccess_main_chain_abs.V10 |
| exposure_HSEBD.V11 | exposure_RDa.V11 | naccess_all_atom_rel.V11 | naccess_main_chain_abs.V11 |
| exposure_HSEBD.V12 | exposure_RDa.V12 | naccess_all_atom_rel.V12 | naccess_main_chain_abs.V12 |
| exposure_HSEBD.V13 | exposure_RDa.V13 | naccess_all_atom_rel.V13 | naccess_main_chain_abs.V13 |
| exposure_HSEBD.V14 | exposure_RDa.V14 | naccess_all_atom_rel.V14 | naccess_main_chain_abs.V14 |
| exposure_HSEBD.V15 | exposure_RDa.V15 | naccess_all_atom_rel.V15 | naccess_main_chain_abs.V15 |

| feature name | feature name | feature name | feature name |
| --- | --- | --- | --- |
| naccess_main_chain_abs.V16 | naccess_non_polar_rel.V15 | network_Between_Cent.V14 | network_Cocitation.V13 |
| naccess_main_chain_rel.V2 | naccess_non_polar_rel.V16 | network_Between_Cent.V15 | network_Cocitation.V14 |
| naccess_main_chain_rel.V3 | naccess_total_side_abs.V2 | network_Between_Cent.V16 | network_Cocitation.V15 |
| naccess_main_chain_rel.V4 | naccess_total_side_abs.V3 | network_Closen_Cent.V2 | network_Cocitation.V16 |
| naccess_main_chain_rel.V5 | naccess_total_side_abs.V4 | network_Closen_Cent.V3 | network_Constraint.V2 |
| naccess_main_chain_rel.V6 | naccess_total_side_abs.V5 | network_Closen_Cent.V4 | network_Constraint.V3 |
| naccess_main_chain_rel.V7 | naccess_total_side_abs.V6 | network_Closen_Cent.V5 | network_Constraint.V4 |
| naccess_main_chain_rel.V8 | naccess_total_side_abs.V7 | network_Closen_Cent.V6 OFC | network_Constraint.V5 |
| naccess_main_chain_rel.V9 | naccess_total_side_abs.V8 | network_Closen_Cent.V7 | network_Constraint.V6 |
| naccess_main_chain_rel.V10 | naccess_total_side_abs.V9; OFC | network_Closen_Cent.V8; OFC FINAL | network_Constraint.V7 |
| naccess_main_chain_rel.V11 | naccess_total_side_abs.V10 | network_Closen_Cent.V9 OFC | network_Constraint.V8 |
| naccess_main_chain_rel.V12 | naccess_total_side_abs.V11 | network_Closen_Cent.V10 ;OFC | network_Constraint.V9 ;OFC |
| naccess_main_chain_rel.V13 | naccess_total_side_abs.V12 | network_Closen_Cent.V11 ;OFC | network_Constraint.V10 |
| naccess_main_chain_rel.V14 | naccess_total_side_abs.V13 | network_Closen_Cent.V12 ;OFC | network_Constraint.V11 |
| naccess_main_chain_rel.V15 | naccess_total_side_abs.V14 | network_Closen_Cent.V13; OFC | network_Constraint.V12 |
| naccess_main_chain_rel.V16 | naccess_total_side_abs.V15 | network_Closen_Cent.V14 | network_Constraint.V13 |
| naccess_non_polar_abs.V2 | naccess_total_side_abs.V16 | network_Closen_Cent.V15 | network_Constraint.V14 |
| naccess_non_polar_abs.V3 | naccess_total_side_rel.V2 | network_Closen_Cent.V16 | network_Constraint.V15 |
| naccess_non_polar_abs.V4 | naccess_total_side_rel.V3 | network_Cluster_Coef.V2 | network_Constraint.V16 |
| naccess_non_polar_abs.V5 | naccess_total_side_rel.V4 | network_Cluster_Coef.V3 | network_Coreness.V2 |
| naccess_non_polar_abs.V6 | naccess_total_side_rel.V5 | network_Cluster_Coef.V4 | network_Coreness.V3 |
| naccess_non_polar_abs.V7 | naccess_total_side_rel.V6 | network_Cluster_Coef.V5 | network_Coreness.V4 |
| naccess_non_polar_abs.V8 | naccess_total_side_rel.V7 | network_Cluster_Coef.V6 | network_Coreness.V5 |
| naccess_non_polar_abs.V9; OFC FINAL | naccess_total_side_rel.V8 | network_Cluster_Coef.V7 | network_Coreness.V6 |
| naccess_non_polar_abs.V10 | naccess_total_side_rel.V9; OFC | network_Cluster_Coef.V8 | network_Coreness.V7 |
| naccess_non_polar_abs.V11 | naccess_total_side_rel.V10 | network_Cluster_Coef.V9; OFC | network_Coreness.V8 |
| naccess_non_polar_abs.V12 | naccess_total_side_rel.V11 | network_Cluster_Coef.V10 | network_Coreness.V9 |
| naccess_non_polar_abs.V13 | naccess_total_side_rel.V12 | network_Cluster_Coef.V11 | network_Coreness.V10 |
| naccess_non_polar_abs.V14 | naccess_total_side_rel.V13 | network_Cluster_Coef.V12 | network_Coreness.V11 |
| naccess_non_polar_abs.V15 | naccess_total_side_rel.V14 | network_Cluster_Coef.V13 | network_Coreness.V12 |
| naccess_non_polar_abs.V16 | naccess_total_side_rel.V15 | network_Cluster_Coef.V14 | network_Coreness.V13 |
| naccess_non_polar_rel.V2 | naccess_total_side_rel.V16 | network_Cluster_Coef.V15 | network_Coreness.V14 |
| naccess_non_polar_rel.V3 | network_Between_Cent.V2 | network_Cluster_Coef.V16 | network_Coreness.V15 |
| naccess_non_polar_rel.V4 | network_Between_Cent.V3 | network_Cocitation.V2 | network_Coreness.V16 |
| naccess_non_polar_rel.V5 | network_Between_Cent.V4 | network_Cocitation.V3 | network_Cyclic_Coeff.V2 |
| naccess_non_polar_rel.V6 | network_Between_Cent.V5 | network_Cocitation.V4 | network_Cyclic_Coeff.V3 |
| naccess_non_polar_rel.V7 | network_Between_Cent.V6 | network_Cocitation.V5 | network_Cyclic_Coeff.V4 |
| naccess_non_polar_rel.V8 | network_Between_Cent.V7 | network_Cocitation.V6 | network_Cyclic_Coeff.V5 |
| naccess_non_polar_rel.V9; OFC | network_Between_Cent.V8 | network_Cocitation.V7 | network_Cyclic_Coeff.V6 |
| naccess_non_polar_rel.V10 | network_Between_Cent.V9; OFC | network_Cocitation.V8 | network_Cyclic_Coeff.V7 |
| naccess_non_polar_rel.V11 | network_Between_Cent.V10 | network_Cocitation.V9 | network_Cyclic_Coeff.V8 |
| naccess_non_polar_rel.V12 | network_Between_Cent.V11 | network_Cocitation.V10 | network_Cyclic_Coeff.V9; OFC |
| naccess_non_polar_rel.V13 | network_Between_Cent.V12 | network_Cocitation.V11 | network_Cyclic_Coeff.V10 |
| naccess_non_polar_rel.V14 | network_Between_Cent.V13 | network_Cocitation.V12 | network_Cyclic_Coeff.V11 |

| feature name | feature name | feature name | feature name |
| --- | --- | --- | --- |
| network_Cyclic_Coeff.V12 | network_Eigen_Cent.V11 | co_evolution_type_1_Kai.V10 | co_evolution_type_1_MI.V9 |
| network_Cyclic_Coeff.V13 | network_Eigen_Cent.V12 | co_evolution_type_1_Kai.V11 | co_evolution_type_1_MI.V10 |
| network_Cyclic_Coeff.V14 | network_Eigen_Cent.V13 | co_evolution_type_1_Kai.V12 | co_evolution_type_1_MI.V11 |
| network_Cyclic_Coeff.V15 | network_Eigen_Cent.V14 | co_evolution_type_1_Kai.V13 | co_evolution_type_1_MI.V12 |
| network_Cyclic_Coeff.V16 | network_Eigen_Cent.V15 | co_evolution_type_1_Kai.V14 | co_evolution_type_1_MI.V13 |
| network_degree.V2 | network_Eigen_Cent.V16 | co_evolution_type_1_Kai.V15 | co_evolution_type_1_MI.V14 |
| network_degree.V3 | network_Hubscore.V2 | co_evolution_type_1_Kai.V16 | co_evolution_type_1_MI.V15 |
| network_degree.V4 | network_Hubscore.V3 | co_evolution_type_1_MIp.V2 | co_evolution_type_1_MI.V16 |
| network_degree.V5 | network_Hubscore.V4 | co_evolution_type_1_MIp.V3 | co_evolution_type_2_Kai.V2 |
| network_degree.V6 | network_Hubscore.V5 | co_evolution_type_1_MIp.V4 | co_evolution_type_2_Kai.V3 |
| network_degree.V7 | network_Hubscore.V6 | co_evolution_type_1_MIp.V5 | co_evolution_type_2_Kai.V4 |
| network_degree.V8 | network_Hubscore.V7 | co_evolution_type_1_MIp.V6 | co_evolution_type_2_Kai.V5 |
| network_degree.V9 | network_Hubscore.V8 | co_evolution_type_1_MIp.V7 | co_evolution_type_2_Kai.V6 |
| network_degree.V10 | network_Hubscore.V9 | co_evolution_type_1_MIp.V8 | co_evolution_type_2_Kai.V7 |
| network_degree.V11 | network_Hubscore.V10 | co_evolution_type_1_MIp.V9; OFC | co_evolution_type_2_Kai.V8 |
| network_degree.V12 | network_Hubscore.V11 | co_evolution_type_1_MIp.V10 | co_evolution_type_2_Kai.V9 |
| network_degree.V13 | network_Hubscore.V12 | co_evolution_type_1_MIp.V11 | co_evolution_type_2_Kai.V10 |
| network_degree.V14 | network_Hubscore.V13 | co_evolution_type_1_MIp.V12 | co_evolution_type_2_Kai.V11 |
| network_degree.V15 | network_Hubscore.V14 | co_evolution_type_1_MIp.V13 | co_evolution_type_2_Kai.V12 |
| network_degree.V16 | network_Hubscore.V15 | co_evolution_type_1_MIp.V14 | co_evolution_type_2_Kai.V13 |
| network_Eccentrality.V2 | network_Hubscore.V16 | co_evolution_type_1_MIp.V15 | co_evolution_type_2_Kai.V14 |
| network_Eccentrality.V3 | network_Status.V2; OFC FINAL | co_evolution_type_1_MIp.V16 | co_evolution_type_2_Kai.V15 |
| network_Eccentrality.V4 | network_Status.V3 | co_evolution_type_1_MIr.V2 | co_evolution_type_2_Kai.V16 |
| network_Eccentrality.V5 | network_Status.V4 | co_evolution_type_1_MIr.V3 | co_evolution_type_2_MIp.V2 |
| network_Eccentrality.V6 | network_Status.V5; OFC | co_evolution_type_1_MIr.V4 | co_evolution_type_2_MIp.V3 |
| network_Eccentrality.V7 | network_Status.V6; OFC | co_evolution_type_1_MIr.V5 | co_evolution_type_2_MIp.V4 |
| network_Eccentrality.V8 | network_Status.V7 | co_evolution_type_1_MIr.V6 | co_evolution_type_2_MIp.V5 |
| network_Eccentrality.V9 | network_Status.V8; OFC FINAL | co_evolution_type_1_MIr.V7 | co_evolution_type_2_MIp.V6 |
| network_Eccentrality.V10 | network_Status.V9; OFC | co_evolution_type_1_MIr.V8 | co_evolution_type_2_MIp.V7 |
| network_Eccentrality.V11 | network_Status.V10; OFC FINAL | co_evolution_type_1_MIr.V9 | co_evolution_type_2_MIp.V8 |
| network_Eccentrality.V12 | network_Status.V11; OFC | co_evolution_type_1_MIr.V10 | co_evolution_type_2_MIp.V9; OFC |
| network_Eccentrality.V13 | network_Status.V12; OFC | co_evolution_type_1_MIr.V11 | co_evolution_type_2_MIp.V10 |
| network_Eccentrality.V14 | network_Status.V13; OFC | co_evolution_type_1_MIr.V12 | co_evolution_type_2_MIp.V11 |
| network_Eccentrality.V15 | network_Status.V14 | co_evolution_type_1_MIr.V13 | co_evolution_type_2_MIp.V12 |
| network_Eccentrality.V16 | network_Status.V15 | co_evolution_type_1_MIr.V14 | co_evolution_type_2_MIp.V13 |
| network_Eigen_Cent.V2 | network_Status.V16 | co_evolution_type_1_MIr.V15 | co_evolution_type_2_MIp.V14 |
| network_Eigen_Cent.V3 | co_evolution_type_1_Kai.V2 | co_evolution_type_1_MIr.V16 | co_evolution_type_2_MIp.V15 |
| network_Eigen_Cent.V4 | co_evolution_type_1_Kai.V3 | co_evolution_type_1_MI.V2 | co_evolution_type_2_MIp.V16 |
| network_Eigen_Cent.V5 | co_evolution_type_1_Kai.V4 | co_evolution_type_1_MI.V3 | co_evolution_type_2_MIr.V2 |
| network_Eigen_Cent.V6 | co_evolution_type_1_Kai.V5 | co_evolution_type_1_MI.V4 | co_evolution_type_2_MIr.V3 |
| network_Eigen_Cent.V7 | co_evolution_type_1_Kai.V6 | co_evolution_type_1_MI.V5 | co_evolution_type_2_MIr.V4 |
| network_Eigen_Cent.V8 | co_evolution_type_1_Kai.V7 | co_evolution_type_1_MI.V6 | co_evolution_type_2_MIr.V5 |
| network_Eigen_Cent.V9 | co_evolution_type_1_Kai.V8 | co_evolution_type_1_MI.V7 | co_evolution_type_2_MIr.V6 |
| network_Eigen_Cent.V10 | co_evolution_type_1_Kai.V9 | co_evolution_type_1_MI.V8 | co_evolution_type_2_MIr.V7 |

| feature name |
| --- |
| co_evolution_type_2_MIr.V8 |
| co_evolution_type_2_MIr.V9 |
| co_evolution_type_2_MIr.V10 |
| co_evolution_type_2_MIr.V11 |
| co_evolution_type_2_MIr.V12 |
| co_evolution_type_2_MIr.V13 |
| co_evolution_type_2_MIr.V14 |
| co_evolution_type_2_MIr.V15 |
| co_evolution_type_2_MIr.V16 |
| co_evolution_type_2_MI.V2 |
| co_evolution_type_2_MI.V3 |
| co_evolution_type_2_MI.V4 |
| co_evolution_type_2_MI.V5 |
| co_evolution_type_2_MI.V6 |
| co_evolution_type_2_MI.V7 |
| co_evolution_type_2_MI.V8 |
| co_evolution_type_2_MI.V9; OFC FINAL |
| co_evolution_type_2_MI.V10 |
| co_evolution_type_2_MI.V11 |
| co_evolution_type_2_MI.V12 |
| co_evolution_type_2_MI.V13 |
| co_evolution_type_2_MI.V14 |
| co_evolution_type_2_MI.V15 |
| co_evolution_type_2_MI.V16 |
