# Supplementary material for: FunSAV: Predicting the Functional Effect of Single Amino Acid Variants Using a Two-Stage Random Forest Model
Source: PLoS One. 2012 Aug 24;7(8):e43847. doi: 10.1371/journal.pone.0043847 (PMC3427247; doi:10.1371/journal.pone.0043847)
Supplement: Table S2 — The mean values and standard deviations of the 15 final selected optimal features for the disease-associated and neutral SAVs. Mean: mean value; SD: standard deviation. P-value was calculated using the unpaired two-sample t-test. (DOC) [file pone.0043847.s003.doc]

**Table S2.** **The mean values and standard deviations of the 15 final selected optimal features for the disease-associated and neutral SAVs.** Mean: mean value; SD: standard deviation. P-value was calculated using the unpaired two-sample *t*-test.

| **Feature name** | **Position** | **Disease-associated SAVs** | | **Neutral SAVs** | | **P-value** |
| --- | --- | --- | --- | --- | --- | --- |
|  |  | **Mean** | **SD** | **Mean** | **SD** |  |
| NACCESS_non_polar_abs | V8 | 14.432 | 21.346 | 33.064 | 28.454 | <2.2e-16 |
| conserve_score | V8 | 2.331 | 1.096 | 2.929 | 0.891 | <2.2e-16 |
| SSpro_code | V8 | 3.133 | 3.829 | 6.101 | 4.462 | <2.2e-16 |
| MW_change | - | 3.977 | 42.383 | -0.0422 | 32.292 | 0.02893 |
| PSSM | V160 | -1.543 | 2.976 | -1.068 | 2.348 | 2.95e-4 |
| B_factor | V7 | 23.341 | 13.955 | 29.699 | 17.667 | 1.04e-15 |
| co_evolution_type_2_MI | V8 | 0.196 | 0.159 | 0.242 | 0.224 | 2.35e-6 |
| exposure_HSEBD | V8 | 21.456 | 9.271 | 14.431 | 8.798 | <2.2e-16 |
| exposure_RD | V8 | 4.894 | 5.788 | 3.628 | 5.933 | 1.134e-5 |
| exposure_HSEBU | V9 | 22.851 | 7.683 | 17.903 | 7.214 | <2.2e-16 |
| exposure_CN | V9 | 40.915 | 13.185 | 34.444 | 11.841 | <2.2e-16 |
| network_Status | V1 | 6.233 | 2.099 | 6.895 | 2.720 | 3.69e-8 |
| network_Closen_Cent | V7 | 0.167 | 0.0478 | 0.151 | 0.0475 | 1.50e-11 |
| network_Status | V9 | 6.305 | 1.816 | 7.112 | 2.446 | 5.243e-14 |
| network_Status | V7 | 6.292 | 1.819 | 7.172 | 2.375 | <2.2e-16 |
