# Supplementary material for: FunSAV: Predicting the Functional Effect of Single Amino Acid Variants Using a Two-Stage Random Forest Model
Source: PLoS One. 2012 Aug 24;7(8):e43847. doi: 10.1371/journal.pone.0043847 (PMC3427247; doi:10.1371/journal.pone.0043847)
Supplement: Table S3 — The prediction performance of RF-based classifiers based on different feature group combinations according to the MDGI Z-Score. (DOC) [file pone.0043847.s004.doc]

**Table S3.** **The prediction performance of RF-based classifiers based on different feature group combinations according to the MDGI Z-Score.**

| **Feature group** | **Dimensionality** | **Abbreviation** | **Performance** |  |  |  |  |
| --- | --- | --- | --- | --- | --- | --- | --- |
|  |  |  | **MCC** | **ACC** | **SEN** | **SPE** | **PRE** |
| All features | 1804 | OFC_ALL | 0.464 | 0.732 | 0.752 | 0.711 | 0.737 |
| MDGI Z-Score>0 | 676 | OFC-0 | 0.464 | 0.732 | 0.751 | 0.712 | 0.738 |
| MDGI Z-Score>1.0 | 65 | OFC-1 | 0.458 | 0.730 | 0.749 | 0.709 | 0.735 |
| MDGI Z-Score>1.5 | 44 | OFC-1.5 | 0.442 | 0.722 | 0.741 | 0.700 | 0.728 |
| MDGI Z-Score >2.0 | 31 | OFC-2 | 0.411 | 0.706 | 0.717 | 0.694 | 0.717 |
| MDGI Z-Score >2.5 | 27 | OFC-2.5 | 0.408 | 0.705 | 0.717 | 0.691 | 0.715 |
| MDGI Z-Score >3.0 | 22 | OFC-3 | 0.399 | 0.700 | 0.722 | 0.676 | 0.707 |
| MDGI Z-Score >3.5 | 19 | OFC-3.5 | 0.400 | 0.701 | 0.725 | 0.675 | 0.706 |
| MDGI Z-Score >4.5 | 18 | OFC-4.5 | 0.396 | 0.699 | 0.722 | 0.674 | 0.705 |
| Final optimal features | 15 | OFC-s | 0.510 | 0.755 | 0.765 | 0.745 | 0.755 |
