# Supplementary material for: FunSAV: Predicting the Functional Effect of Single Amino Acid Variants Using a Two-Stage Random Forest Model
Source: PLoS One. 2012 Aug 24;7(8):e43847. doi: 10.1371/journal.pone.0043847 (PMC3427247; doi:10.1371/journal.pone.0043847)
Supplement: Table S4 — The importance and contributeon of the 15 final optimal features by removal or inclusion of this feature to the first-stage RF classifier. (DOC) [file pone.0043847.s005.doc]

**Table S4.** **The importance and contribution of the 15 final optimal features by removal or inclusion of this feature to the first-stage RF classifier.**

| **Features name** | **MCC decrease^A^** | **Feature removal** |  |  |  |  | **Feature only^B^** |  |  |  |  |
| --- | --- | --- | --- | --- | --- | --- | --- | --- | --- | --- | --- |
|  |  | **MCC** | **ACC** | **SEN** | **SPE** | **PRE** | **MCC** | **ACC** | **SEN** | **SPE** | **PRE** |
| NACCESS_non_polar_abs.V8 | 0.019 | 0.491 | 0.746 | 0.756 | 0.735 | 0.755 | 0.227 | 0.615 | 0.654 | 0.573 | 0.623 |
| conserve_score_win.V8 | 0.014 | 0.496 | 0.748 | 0.756 | 0.740 | 0.758 | 0.147 | 0.575 | 0.607 | 0.539 | 0.587 |
| SSpro_code.V8 | 0.036 | 0.474 | 0.737 | 0.745 | 0.730 | 0.748 | 0.337 | 0.669 | 0.763 | 0.567 | 0.655 |
| MW_change | 0.03 | 0.480 | 0.741 | 0.750 | 0.730 | 0.750 | 0.267 | 0.631 | 0.577 | 0.689 | 0.667 |
| co_evolution_type_2_MI.V8 | 0.016 | 0.494 | 0.747 | 0.758 | 0.736 | 0.756 | 0.167 | 0.582 | 0.539 | 0.628 | 0.610 |
| PSSM.V160 | 0.021 | 0.489 | 0.745 | 0.750 | 0.739 | 0.756 | 0.119 | 0.552 | 0.422 | 0.693 | 0.598 |
| B-factor.V7 | 0.018 | 0.492 | 0.746 | 0.754 | 0.739 | 0.757 | 0.059 | 0.530 | 0.534 | 0.525 | 0.548 |
| exposure_HSEBD.V8 | 0.019 | 0.491 | 0.746 | 0.758 | 0.733 | 0.754 | 0.329 | 0.663 | 0.632 | 0.697 | 0.692 |
| exposure_RD.V8 | 0.002 | 0.508 | 0.754 | 0.764 | 0.745 | 0.764 | 0.173 | 0.582 | 0.495 | 0.676 | 0.622 |
| exposure_HSEBU.V9 | 0.004 | 0.506 | 0.753 | 0.763 | 0.743 | 0.762 | 0.261 | 0.631 | 0.645 | 0.616 | 0.644 |
| exposure_CN.V9 | 0.007 | 0.503 | 0.752 | 0.762 | 0.740 | 0.760 | 0.226 | 0.613 | 0.605 | 0.621 | 0.633 |
| network_Status.V7 | 0.001 | 0.509 | 0.755 | 0.764 | 0.745 | 0.764 | 0.081 | 0.541 | 0.542 | 0.539 | 0.560 |
| network_Status.V1 | 0.018 | 0.492 | 0.746 | 0.760 | 0.732 | 0.754 | 0.138 | 0.570 | 0.603 | 0.535 | 0.583 |
| network_Closen_Cent.V7 | 0.001 | 0.509 | 0.755 | 0.763 | 0.747 | 0.765 | 0.081 | 0.541 | 0.542 | 0.539 | 0.560 |
| network_Status.V9 | 0.01 | 0.500 | 0.750 | 0.761 | 0.739 | 0.759 | 0.101 | 0.551 | 0.568 | 0.533 | 0.568 |
| Network group | 0.05 | 0.460 | 0.731 | 0.756 | 0.703 | 0.734 | 0.187 | 0.595 | 0.637 | 0.549 | 0.604 |
| Exposure group | 0.037 | 0.473 | 0.737 | 0.753 | 0.719 | 0.743 | 0.318 | 0.660 | 0.678 | 0.639 | 0.670 |

^A^ MCC decrease of the trained classifier by removal of the feature;

^B^ Performance of the trained classifier using the individual feature only.
