# Supplementary material for: FunSAV: Predicting the Functional Effect of Single Amino Acid Variants Using a Two-Stage Random Forest Model
Source: PLoS One. 2012 Aug 24;7(8):e43847. doi: 10.1371/journal.pone.0043847 (PMC3427247; doi:10.1371/journal.pone.0043847)
Supplement: Table S5 — Performance of the first-stage and two-stage classifiers based on an independent test dataset. (DOC) [file pone.0043847.s006.doc]

**Table S5. Performance of the first-stage and two-stage classifiers based on an independent test dataset.**

| **Feature group** | **Performance** | | | | | |
| --- | --- | --- | --- | --- | --- | --- |
|  | **MCC** | **ACC** | **SEN** | **SPE** | **PRE** | **AUC** |
| SNAP | 0.365 | 0.631 | 0.934 | 0.377 | 0.556 | 0.764 |
| SIFT | 0.475 | 0.728 | 0.828 | 0.644 | 0.660 | 0.788 |
| PolyPhen2 | 0.479 | 0.720 | 0.877 | 0.589 | 0.641 | 0.809 |
| nsSNPAnalyzer | 0.264 | 0.638 | 0.525 | 0.733 | 0.621 | 0.629 |
| PANTHER | 0.412 | 0.694 | 0.811 | 0.596 | 0.627 | 0.791 |
| PhD-SNP | 0.440 | 0.720 | 0.730 | 0.712 | 0.679 | 0.721 |
| First-stage classifier | 0.482 | 0.739 | 0.779 | 0.705 | 0.688 | 0.793 |
| PolyPhen2+SIFT+SNAP  +nsSNPAnalyzer+PANTHER+PhD-SNP | 0.546 | 0.769 | 0.828 | 0.719 | 0.711 | 0.834 |
| Two-stage classifier | 0.627 | 0.810 | 0.895 | 0.722 | 0.763 | 0.873 |
