# Supplementary material for: FunSAV: Predicting the Functional Effect of Single Amino Acid Variants Using a Two-Stage Random Forest Model
Source: PLoS One. 2012 Aug 24;7(8):e43847. doi: 10.1371/journal.pone.0043847 (PMC3427247; doi:10.1371/journal.pone.0043847)
Supplement: Table S6 — Analysis of several important final features that are related to the residue locations in three exemplar proteins in the case study. (DOC) [file pone.0043847.s007.doc]

**Table S6.** Analysis of several important final selected features that are related to the residue locations in three exemplar proteins in the case study.

| Protein | ID | Variant | NAC_npa_V8 | SSpro_V8 | HSEBD_V8 | HSEBU_V9 | RD_V8 | CN_V9 |
| --- | --- | --- | --- | --- | --- | --- | --- | --- |
| hATR | 2IDX | M239K | 142.7 | exposed | 8 | 17 | 1.99 | 25 |
| hATR | 2IDX | I96T | 0 | buried | 30 | 21 | 6.94 | 54 |
| hATR | 2IDX | R191W | 2.76 | buried | 28 | 24 | 6.75 | 55 |
| PAF-AH | 3D59 | V279F | 0 | buried | 32 | 35 | 10.87 | 66 |
| PAF-AH | 3D59 | Q281R | 0 | buried | 29 | 21 | 5.27 | 56 |
| PAF-AH | 3D59 | R92H | 22.95 | exposed | 13 | 20 | 2.89 | 42 |
| Noggin | 1M4U | R204L | 17.18 | buried | 12 | 17 | 2.64 | 38 |
